# Interfacial Microenvironment Effects at Laser-Made Gold Nanoparticles Steer Carbon Dioxide Reduction Product Generation

**DOI:** 10.1021/acsmaterialsau.4c00161

**Published:** 2025-04-29

**Authors:** Connor P. Cox, Qishen Lyu, Madeleine K. Wilsey, Likun Cai, Lydia R. Schultz, Jason R. Maher, Astrid M. Müller

**Affiliations:** † Materials Science Program, University of Rochester, Rochester, New York 14627, United States; ‡ Department of Chemical Engineering, University of Rochester, Rochester, New York 14627, United States; § Department of Chemistry, University of Rochester, Rochester, New York 14627, United States

**Keywords:** electrocatalysis, carbon dioxide reduction, syngas, gold nanoparticles, pulsed laser in liquid synthesis, self-assembled monolayers, microenvironment

## Abstract

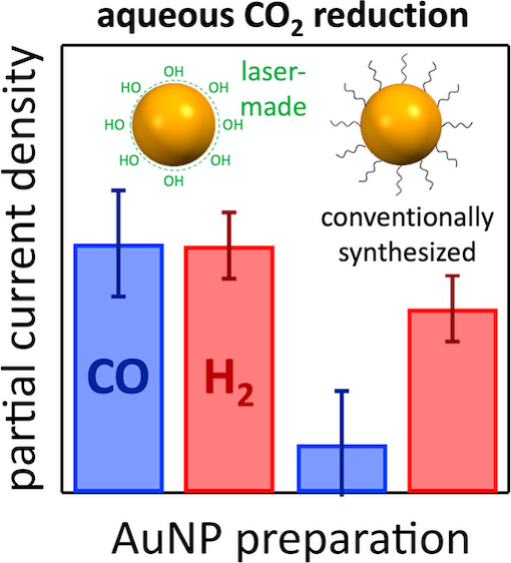

This study emphasizes the critical importance of using surfactant-free gold nanoparticles to gain mechanistic insights and improve the energy efficiency and carbon monoxide selectivity in aqueous carbon dioxide reduction electrocatalysis. We utilized pulsed laser in liquid synthesis to prepare surfactant-free gold nanoparticles with a nonequilibrium cauliflower morphology, which demonstrated superior catalytic performance compared to conventionally synthesized citrate-capped gold nanoparticles. By functionalizing gold nanoparticles with nine *n*-alkanethiols and two nitrogen-containing thiols, we investigated how the chemical identity of interfacial ligands and their corresponding self-assembled monolayers (SAMs) influence the selectivity and activity of gold nanoparticle-catalyzed CO_2_ reduction. This approach enabled a detailed understanding of how SAM characteristics at gold nanocatalyst interfaces affect key aspects of CO_2_ electrocatalysis, including CO_2_ mass transport and interfacial water behavior. The laser-synthesized gold nanoparticles exhibited improved performance across all surface modifications. Our findings highlight the significance of precise control over material surfaces in understanding catalyst microenvironments, which is essential for optimizing CO_2_ reduction processes and forming a foundation for sustainable syngas production through tailored nanomaterial design and functionalization strategies.

## 1 Introduction

A low-carbon economy requires the advancement of sustainable chemical manufacturing processes that reduce CO_2_ emissions.^1^ Electrocatalytic reduction of CO_2_ is emerging as a promising method for producing valuable chemicals.^2^ Electrocatalysis offers several advantages: it operates under ambient conditions, can be implemented in distributed units, does not require chemical reductants, is globally scalable, and can be sustainably powered by renewable electricity.^1^ To facilitate the transition to a decarbonized economy, it is crucial to quantitatively understand materials interfaces, catalyst microenvironments and how selectivity for specific desired products arises. This knowledge will accelerate the design and development of advanced catalytic materials and processes.^2^

Gold cathodes have been identified as effective catalysts for the reduction of CO_2_ to CO, H_2_, and small quantities of formate.^3−5^ The gases H_2_ and CO form the primary components of synthesis gas (syngas), a critical intermediate in chemical manufacturing, including Fischer–Tropsch synthesis.^6−8^ Currently, syngas is predominantly produced through energy-intensive steam reforming of fossil sources, resulting in syngas with varying CO-to-H_2_ compositions and significant amounts of contaminants, which requires purification prior to downstream catalytic processes.^9^ In contrast, syngas generated via electrocatalytic CO_2_ reduction is inherently clean, as it produces the gases H_2_ and CO only, thereby obviating the need for purification steps, which further lowers energy demands.^9^ Precise control of the CO-to-H_2_ ratio is essential for the targeted downstream catalytic reactions.^6−8^ Hydrogen evolution competes with CO production because CO_2_ reduction reactions require a proton source, and H_2_ and CO evolution have standard potentials that differ by only 100 mV at pH 6.8, which was used here. Furthermore, the mass transport of protons is faster than that of CO_2_.^2^ Therefore, directing aqueous CO_2_ reduction to CO is a challenge.

Here, we report how control of the chemical identity of interfacial molecules at gold nanoparticles (AuNPs) immobilized on hydrophilic carbon fiber paper (hCFP)^10^ cathodes steers the CO-to-H_2_ ratio in electrocatalytic aqueous CO_2_ reduction in an H-cell. The chemical identity of interfacial molecules influences the catalyst microenvironment, defined as the region of the electrolyte adjacent to the electrode that is affected by the electric field of the applied potential.^11^ We used surfactant-free AuNPs and compared them to commercial citrate-capped AuNPs of similar size, conventionally prepared by nucleation growth, which we subjected to an electroreduction step aimed at surface citrate removal.^9^ We prepared the surfactant-free AuNPs by pulsed laser in liquid synthesis. The laser method enables exceptional control of the chemical identity of interfacial molecules at nanomaterials and does not require surfactants, providing electrocatalyst microenvironments unencumbered by surfactant molecules.^12^ In contrast, conventional synthetic routes need surfactants for size control.^12^ The presence of surfactants alters nanomaterial surfaces, complicates reaction mechanisms and their understanding, and often lowers catalytic performance.^12^ In pulsed laser in liquid synthesis, nanoparticle size control arises from the light–matter interactions.^12^ The role of surfactants in electrocatalysis is often underappreciated. In previous work, we found that conventionally synthesized, citrate-capped AuNPs led to 5.6 times more unwanted H_2_ instead of CO during aqueous CO_2_ reduction than analogous AuNPs that were electroreduced to remove citrate.^9^ Surfactant removal is often incomplete because of entropic penalties. Therefore, we used here inherently citrate-free AuNPs that we obtained from pulsed laser in liquid synthesis of a solid gold target in water, to prevent ill-defined surface conditions in the catalyst microenvironment. In addition to nanomaterial synthesis without surfactants, pulsed laser in liquid synthesis provides sufficient energy and rapid cooling to enable the preparation of nonequilibrium nanomaterials by accessing extreme phase space regions, which are difficult to synthesize by conventional chemical routes.^12^ As a consequence, laser-made nanomaterials with surface facets that differed from analogous equilibrium nanomaterials have been reported.^12^ Surface-facet-dependent CO_2_ reduction selectivity has been observed.^13^ In contrast, conventional synthesis by nucleation growth generates equilibrium-structure AuNPs.^14,15^

In addition to studying bare laser-made or citrate-capped–electroreduced AuNPs, we functionalized them with nine *n*-alkanethiols, CH_3_(CH_2_)*_n_*SH (*n* = 3–11), that formed thiolate self-assembled monolayers (SAMs) and two nitrogen-containing thiols, 4-mercaptopyridine, or 2,5-dimercapto-1,3,4-thiadiazole, to gain a deeper understanding and reveal how the alkane chain length and basicity of the organic thiols affected the catalytic activity and CO-to-H_2_ ratio of AuNP-catalyzed CO_2_ reduction electrocatalysis. The reduction of CO_2_ to value-added products is facilitated by suppressing competing H_2_ evolution, achieved by minimizing interfacial water, along with enhancing CO_2_ mass transport to the catalyst.^9^ Therefore, the hydrophobicity of interfacial molecules is critical. The methylene chain length of *n*-alkanethiols influences the lateral ordering of SAMs on gold surfaces,^16^ which in turn affects the binding preferences on AuNPs (e.g., planes, edges, corners), as well as the interchain hydrophobicity.^17^ Increased hydrophobicity reduces surface water and enhances local CO_2_ concentrations. Electrochemical CO_2_ adsorption limits CO production in CO_2_ reduction at gold.^18^ We investigated nine *n*-alkanethiols, CH_3_(CH_2_)*_n_*SH (*n* = 3–11), to identify trends in interfacial characteristics and correlate them with CO_2_ reduction performance. Additionally, two nitrogen-containing thiols were investigated, as the local basicity of surface amines increases the interfacial pH, which can suppress H_2_ evolution and promote CO_2_ adsorption.^19^

## 2 Results and Discussion

### 2.1 AuNP–hCFP Cathodes

**Figure 1:**
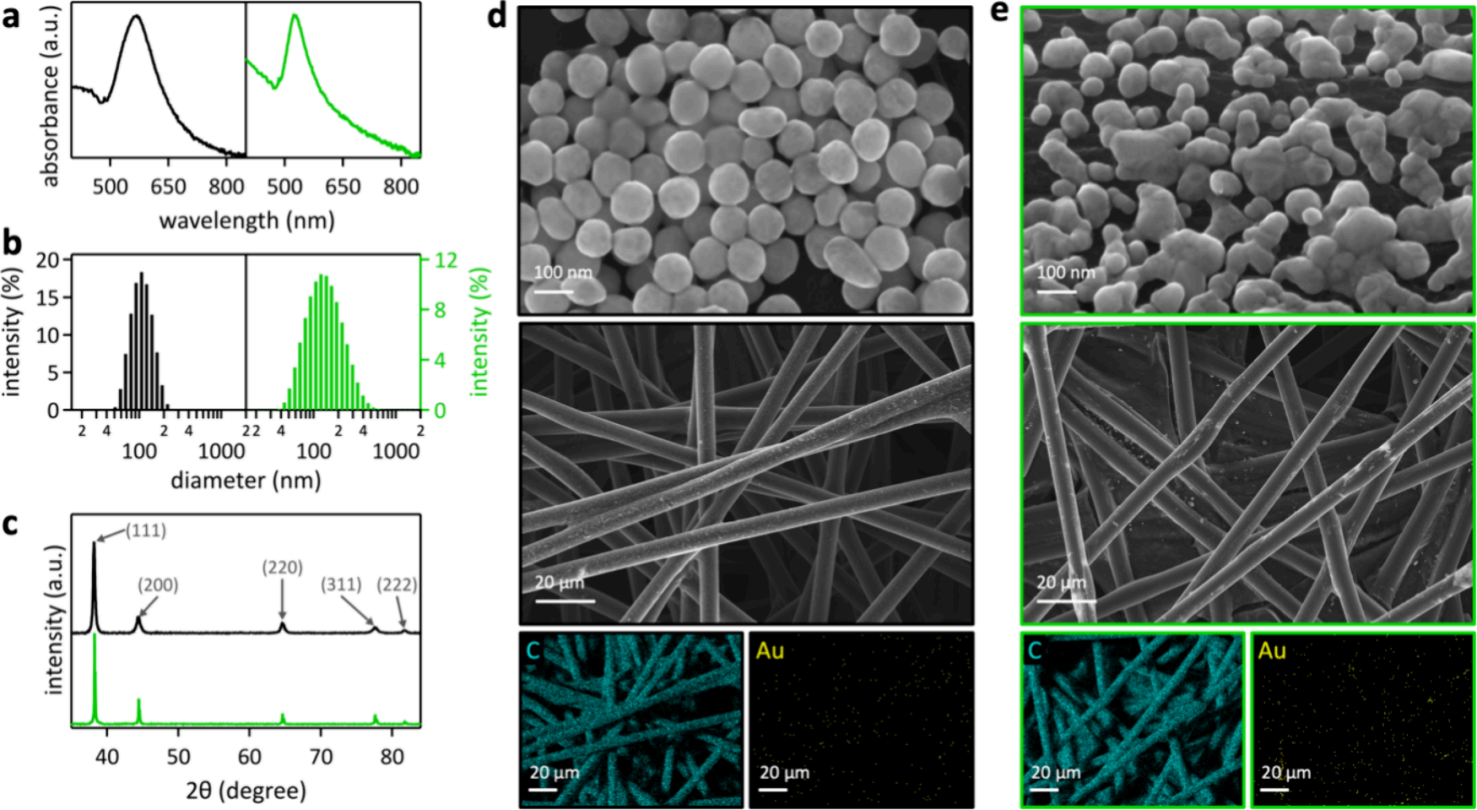
(a) Optical spectra, (b) DLS data, (c) XRD data with Miller indices of aqueous colloids of conventionally synthesized citrate-capped (black) and laser-made surfactant-free AuNPs (green). SEM images of (d) conventionally synthesized citrate-capped–electroreduced and (e) laser-made surfactant-free AuNPs.

We compared laser-made surfactant-free AuNPs to commercial conventionally synthesized citrate-capped AuNPs. Surfactant-containing AuNPs have extensively been used to study CO_2_ reduction.^20−25^ We used optical spectroscopy, dynamic light scattering (DLS), and scanning electron microscopy (SEM) imaging to characterize the size of AuNPs. The optical spectra of laser-made or conventionally synthesized, citrate-capped colloidal AuNPs had maxima at 529.6 ± 0.2 or 566.5 ± 0.1 nm, with Gaussian peak widths of 29.2 ± 0.5 or 56.2 ± 0.6 nm, respectively (Figure 1a). The surface plasmon resonance of AuNPs gives rise to a broad absorption band around 520–580 nm, depending on AuNP size.^26^ Larger AuNPs exhibit increased scattering and significantly broader peaks that appear at longer wavelengths because larger AuNPs have larger optical cross sections and a larger albedo, which is the ratio of scattering to total extinction.^26−29^ The ratio of scattering to absorption is highly dependent on the dimensions of the AuNPs, approaching unity for spherical AuNPs with diameters around 100 nm,^30^ as used in this study. In contrast, AuNPs smaller than 40 nm exhibit optical spectra dominated by absorption.^30^ Consequently, the optical spectra of the AuNPs in this work contain approximately equal contributions from both absorption and scattering. DLS data show that pulsed laser in liquid synthesis enabled the preparation of citrate-free, similar sized AuNPs size as the commercial, conventionally synthesized, citrate-capped AuNPs (Figure 1b). The laser-made AuNPs were slightly larger (116 ± 4 nm) than the commercial AuNPs (102 ± 1 nm). Our observation of a blue-shifted, narrower band in the optical spectrum of laser-made AuNPs, compared to conventionally synthesized, citrate-capped AuNPs, along with the slightly larger size of laser-made AuNPs suggests that the laser-made AuNPs scattered less light than the conventionally synthesized, citrate-capped AuNPs. Note that the larger size of laser-made AuNPs is not an issue for the evaluation of the CO_2_ reduction mass activity for CO formation here because larger AuNPs have been found to be inferior CO_2_ reduction catalysts,^31^ especially AuNPs > 10 nm,^32^ so that we err on the side of underestimating the benefits of pulsed laser in liquid synthesis for AuNP preparation.

Laser-made AuNPs, synthesized from a gold foil target in water, did not contain any surfactants. Nevertheless, they remained colloidal in water because of their high zeta potential of −45.5 ± 0.9 mV. X-ray diffraction (XRD) data of conventionally synthesized and laser-made AuNPs show that pulsed laser in liquid synthesis produced nonequilibrium nanoparticles that exhibited a different faceting than AuNPs that were conventionally synthesized by nucleation growth (Figure 1c). We normalized the XRD patterns to the (111) peak and found that the (200) or (311) peak maxima of laser made AuNPs were 1.5 or 1.6 times higher than that of analogous conventionally prepared AuNPs, respectively (Figure 1c). The (220) and (222) peak heights did not change as a function of AuNP synthesis method.

The conventionally synthesized citrate-capped–electroreduced AuNPs exhibited spherical shape with 100 nm diameter (Figure 1d). The laser-made AuNPs had approximately 115 nm diameter, comprised of smaller spheres that were fused together (Figure 1e), consistent with DLS data (Figure 1b). Pulsed laser ablation in liquid synthesis typically yields AuNPs with sizes in the range of 5–40 nm.^12^ The significantly larger particle size observed in this work suggests the involvement of additional mechanisms, such as postsynthesis agglomeration and laser-induced melting^33−35^ during the 1-h pulsed laser irradiation used here. We propose that repeated exposure of already-formed particles to the laser beam promotes coalescence and reshaping, resulting in the formation of larger, fused nanoparticles. The formation of agglomerated AuNPs through combined ablation–melting processes has been reported.^36−38^ While a detailed investigation of this mechanism is beyond the scope of the current study, the particle size and morphology observed here are more consistent with a process involving both laser ablation and in-solution melting, rather than pure ablation alone. The laser-induced fusing created concave edges at the fusing boundaries (Figure 1e). Assembly of AuNPs by nanosecond laser pulses in liquids has been reported.^39^ Similar concave edges occur in twinning. Twinned small AuNPs have been found to be favorable for CO_2_ reduction to CO because of an increased number of undercoordinated surface sites.^40^ Strain-engineering modulated the electronic structure of small AuNPs and enhanced CO_2_ reduction to CO.^41^ Twinning of large AuNPs by Cu^2+^-mediated Ostwald ripening has been reported,^42^ but the presence of copper would complicate CO_2_ reduction product analysis, as copper is the premier CO_2_ reduction catalyst for the production of hydrocarbons and oxygenates.^43^

Integrated AuNP–hCFP cathodes were prepared to electrocatalyze aqueous CO_2_ reduction because nanocatalysts need electrode supports. Carbon fiber paper is an electrically conducting, inexpensive, globally scalable, nontoxic, robust carbon support material^10^ with high overpotential for CO_2_ reduction^9^ and a high carbon surface area of 468 cm^2^ per geometric cm^2^.^44^ The high surface area enhances electrocatalytic product generation, which is needed for quantification of these products without large uncertainty. Integrated cathodes were prepared by immersing hCFP in aqueous AuNP colloids of commercial, conventionally synthesized, citrate-capped or laser-made surfactant-free AuNPs. Citrate-capped AuNP–hCFP electrodes were subjected to an electroreduction step aimed at removing surface citrate, following a previously reported protocol.^9^ Integrated conventionally synthesized–electroreduced and laser-made AuNP–hCFP electrodes exhibited uniform distributions of the AuNPs on carbon fibers, penetrating deep into the carbon fiber network, demonstrated by SEM images and EDX data (Figure 1d,e). Inductively coupled plasma mass spectrometry (ICP-MS) was used to quantify the gold content of AuNP–hCFP electrodes by digesting the samples in aqua regia. This analysis validated the gold mass loading across differently prepared electrodes. For each preparation, three electrodes were analyzed, with three ICP-MS measurements conducted per electrode. The results showed no significant difference in gold mass loading between laser-fabricated AuNP–CFP and citrate-capped, electrochemically reduced AuNP–hCFP modified with selected thiol surfactants (Figure S1). From these data, we determined an average gold loading of 8.9 μg cm^–2^_geometric_. Additionally, negligible amounts of gold were detected in the Teflon tub following the immersion-based composite fabrication (Figure S1), confirming minimal gold loss during the immersion deposition.

**Figure 2:**
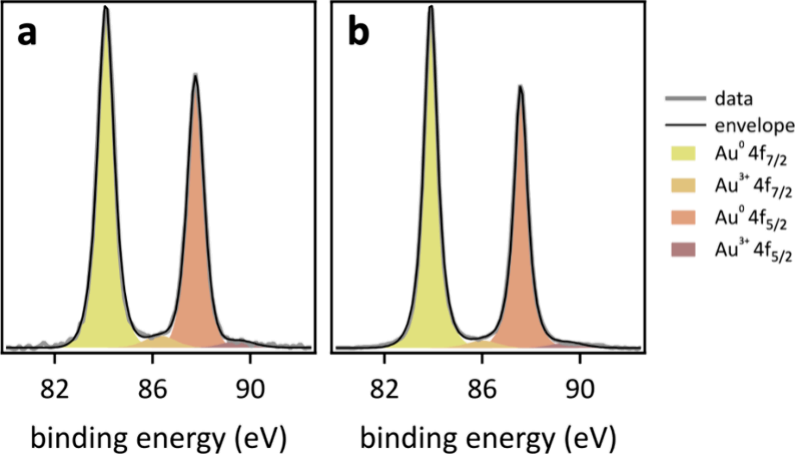
XPS data of (a) conventionally synthesized citrate-capped–electroreduced and (b) laser-made surfactant-free AuNP–hCFP cathodes.

X-ray photoelectron spectroscopy (XPS) data of these integrated electrodes revealed the presence of surface gold, carbon, and oxygen (Figure 2). XPS data of conventionally synthesized citrate-capped–electroreduced AuNPs on hCFP were consistent with previously reported data,^9^ and did not reveal the presence of sodium (from sodium citrate capping molecules), suggesting successful removal of surface citrate at XPS detection levels. The XPS data of laser-made and citrate-capped–electroreduced AuNPs on hCFP showed negligible differences. High resolution Au 4f data were fitted using a Gaussian–Lorentzian doublet with an orbital splitting of (3.5 ± 0.14) eV for Au 4f_7/2_ and Au 4f_5/2_. The doublet peaks were constrained to full width half-maximum (fwhm) values of 0.5–1.5 eV and a peak area ratio of 4:3, in agreement with reported values.^45^ Four component peaks were required to fit the measured data. The predominant species was metallic gold at a binding energy of (84.4 ± 0.1) eV for Au 4f_7/2_ and (88.0 ± 0.1) eV for Au 4f_5/2_.^9^ Additionally, peaks corresponding to the Au^3+^ oxidation state were observed at (87.0 ± 0.2) and (90.5 ± 0.2) eV, attributable to Au 4f_7/2_ and Au 4f_5/2_ of Au_2_O_3_, respectively, which resulted from oxide formation at the AuNP surface upon exposure to air.^46,47^

While the XPS data of conventionally synthesized citrate-capped–electroreduced AuNPs suggested the absence of surface sodium citrate capping ligands within the detection limit of XPS, the complete removal of surfactants is typically unattainable because of entropic penalties. Citrate is known to promote H_2_ production at AuNP cathodes.^48^ Therefore, we compared here the CO_2_ reduction selectivity of conventionally synthesized citrate-capped–electroreduced AuNPs with that of laser-made AuNPs, which are inherently citrate-free.

### 2.2 CO_2_ Reduction at Bare Laser-Made vs Citrate-Capped–Electroreduced AuNPs

**Figure 3:**
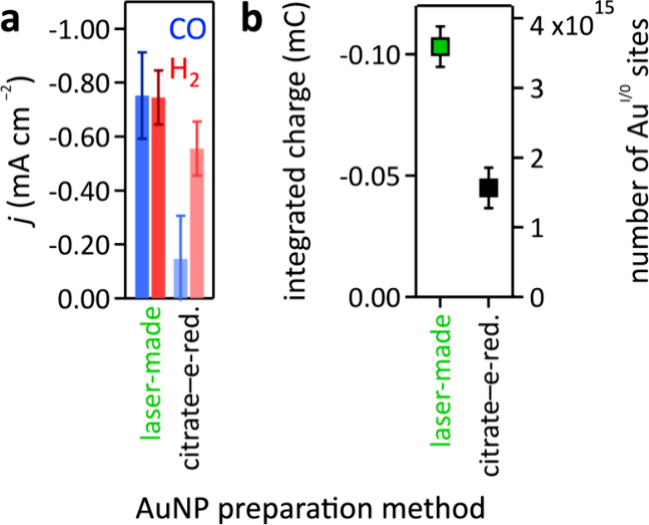
(a) Partial current densities for CO and H_2_ of aqueous CO_2_ reduction at–0.8 V vs RHE and (b) number of electrochemically active gold sites at bare conventionally synthesized citrate-capped–electroreduced or laser-made surfactant-free AuNP–hCFP cathodes.

Electrocatalytic CO_2_ reduction to CO and H_2_ at AuNPs on hCFP cathodes showed that laser made AuNPs produced two times more current density than citrate-capped–electroreduced AuNPs. Additionally, the CO-to-H_2_ ratio at laser-made AuNPs was a factor of 3.8 higher than that of citrate-capped–electroreduced AuNPs (Figure 3a). We used partial current density instead of faradaic efficiency as figure of merit because partial current densities provide information on activity in addition to selectivity; both CO_2_ reduction processes to CO and H_2_ are two-electron–two-proton transformations.

The cathodes prepared with laser-made surfactant-free AuNPs exhibited a factor of 3.8 more electrochemically active surface gold sites than conventionally synthesized citrate-capped–electroreduced AuNP–hCFP cathodes (Figure 3b), derived from cyclic voltammograms shown in the Supporting Information (SI, Figures S2–S4). The observed lower number of accessible Au sites at citrate-capped–electroreduced compared to laser-made AuNPs suggests that citrate removal by electroreduction was incomplete. Additionally, the laser-made AuNPs, albeit slightly larger than the citrate-capped–electroreduced AuNPs, exhibited nonequilibrium faceting and fusing of smaller AuNPs giving rise to cauliflower structures with concave edges at the fusing boundaries (Figure 1), thus providing more undercoordinated sites than the conventionally synthesized equilibrium material, likely contributing to the higher number of electrochemically active gold sites in cathodes with laser-made AuNPs.

### 2.3 SAM–AuNP–hCFP Cathodes

Integrated citrate-capped–electroreduced or laser-made AuNP–hCFP cathodes were functionalized with nine *n*-alkanethiols, CH_3_(CH_2_)*_n_*SH (*n* = 3–11), 4-mercaptopyridine, or 2,5-dimercapto-1,3,4-thiadiazole. We investigated how organic thiolate SAMs on AuNPs steered CO and H_2_ generation, predicated on the key design criteria for aqueous CO_2_ reduction to useful carbon products, i.e., CO in gold-catalyzed CO_2_ reduction. The design criteria are (1) elimination of water at the catalyst surface to suppress the competing H_2_ evolution, (2) enhancement of mass transport of CO_2_ molecules to the catalyst surface, and (3) facilitating the adsorption and bending of CO_2_ at the cathodic catalyst surface upon the first electron transfer, to form the initial surface-bound C–O intermediate.^9^

**Figure 4:**
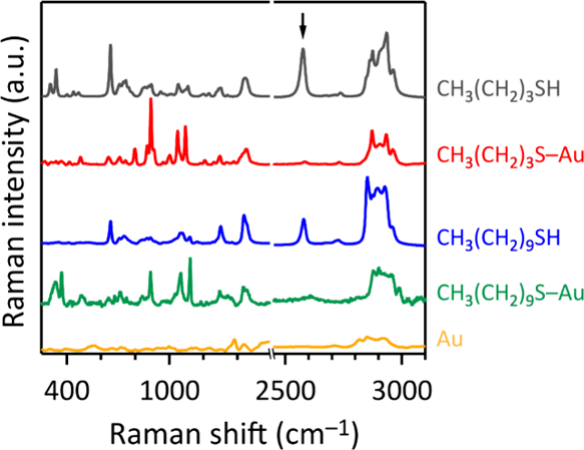
Raman data of aqueous colloids of 1-butanethiol, 1-decanethiol, and respective SAMs on laser-made AuNPs, showing the loss of the S–H vibration at 2575 cm^–1^ (indicated by the arrow) upon Au–S bond formation.

Raman data of select aqueous colloids of *n*-alkanethiolate SAMs on laser-made AuNPs show that the thiols were covalently bound to AuNP surfaces. We were unable to collect Raman spectra of integrated AuNP–hCFP cathodes, presumably because of the low sample density at a catalyst loading of 8.9 μg cm^–2^_geometric_ on hCFP with a high surface area of 468 cm^2^ per geometric cm^2^,^44^ resulting in a catalyst loading with respect to the real surface area of 21 ng cm^–2^. We prepared the aqueous colloids of *n*-alkanethiolate SAMs on laser-made AuNPs by adding 1-butanethiol or 1-decanethiol to aqueous laser-made AuNP colloids, utilizing the same thiol-to-AuNP ratio as used for making integrated cathodes. Raman spectroscopy revealed that this mild treatment of the AuNPs with 1-butanethiol or 1-decanethiol resulted in covalent linkage of gold to the thiols via an Au–S bond, evident from the loss of the S–H vibration in the thiol-containing samples (Figure 4). The Raman spectra of our *n*-alkanethiol functionalized AuNPs are in agreement with spectra reported for analogous *n*-alkanethiol modified gold.^49,50^ More details are in the SI (Figure S5).

Electrochemical impedance spectroscopy (EIS) data of integrated AuNP–hCFP cathodes were collected in the CO_2_ reduction setup, to obtain values that are relevant for this work. Fitting these Nyquist plot impedance data with the Randles-like equivalent circuit model shown in Figure S6a and following a protocol that has been reported for CO_2_ reduction using the same electrochemical cell geometry as the one employed here,^51^ provided resistance values (Figure S6b). The obtained electrolyte resistance values were similar across all preparations and amounted to 13.5 ± 1.2 Ω, in agreement with reported work that used an analogous CO_2_ reduction setup and electrolyte.^52^ The hCFP resistance values were 0.39 ± 0.11 Ω across all preparations, suggesting good electrical contact between AuNPs and the carbon support. The charge transfer resistance of laser-made AuNP–hCFP cathodes was smaller than that of citrate-capped–electroreduced AuNP cathodes, corroborating that electroreduction of citrate-capped AuNPs did not remove all citrate.

For thiol-functionalized AuNPs on hCFP, we focus here on EIS data of cathodes with laser-made AuNPs, since the presence of residual surface citrate complicates the catalyst microenvironment. The observable charge transfer resistance of surface-functionalized cathodes encompasses contributions from the charge transfer from the AuNPs through the surface functionalization molecules, which is governed by the identity and arrangement of the thiolates on the gold surfaces, and from the charge transfer through the electrochemical double layer, which is affected by the identity and concentration of chemical species in the electrochemical double layer, including the concentration of CO_2_.^53^ As a consequence, CO_2_ mass transport effects can obfuscate charge transfer characteristics that were reported for SAMs on gold outside of this electrocatalytic reaction network. Yet, only charge transfer characteristics with CO_2_ present and under CO_2_ reduction conditions can accurately capture the processes in CO_2_ reduction electrocatalysis. The charge transfer resistance values of laser-made AuNPs functionalized with even or odd *n*-alkanethiols decreased as a function of methylene chain length, in contrast to reported work on *n*-alkanethiolate SAMs on bulk gold without CO_2_,^54^ likely because *n*-alkanethiolate SAMs with higher hydrophobicity increased the CO_2_ mass transport and, as a consequence, electron transfer in this CO_2_ reduction system. The charge transfer resistance of cathodes with laser-made AuNPs functionalized with 4-mercaptopyridine was the lowest obtained, consistent with reports that 4-mercaptopyridine binds on gold at near neutral pH such that it lies flat^55,56^ (cf. XPS discussion in Section 2.4), greatly decreasing the electron transfer distance across the surface functionalization, compared to upright thiolates that form SAMs. Modification of laser-made AuNP cathodes with 2,5-dimercapto-1,3,4-thiadiazole led to the highest charge transfer resistance among laser-made AuNP cathodes, consistent with upright, SAM-forming binding of the 1,3,4-thiadiazole-2,5-dithiol tautomer on gold, as reported^57^ and as observed here.

High-resolution Au 4f and S 2p XPS data (Figure S7) gave further insights into the nature of the binding of thiols to AuNPs. High resolution Au 4f spectra collected for thiol modified AuNPs required three Gaussian–Lorentzian doublet peaks. Both, metallic gold and Au_2_O_3_ species were observed, analogous to the bare AuNPs (Figure 2). However, for thiol modified AuNPs, a third doublet was required with peaks at (85.6 ± 0.2) and (89.0 ± 0.1) eV, attributable to Au 4f_7/2_ and Au 4f_5/2_ of Au^+^, respectively, indicative of Au–S.^47^ Across all preparations, the laser made AuNPs showed a higher Au–S content than the conventionally synthesized citrate-capped–electroreduced AuNPs. High resolution S 2p XPS data showed up to three different sulfur species at the surface. These peaks were fitted using a Gaussian–Lorentzian doublet with an orbital splitting of (1.2 ± 0.06) eV for S 2p_3/2_ and S 2p_1/2_, with a constrained 2:1 peak ratio, using a uniform fwhm of (1.0 ± 0.4) eV for all peaks.^58,59^ The doublet with binding energies of (162.4 ± 0.2) and (163.5 ± 0.3) eV for S 2p_3/2_ and S 2p_1/2_, respectively, is consistent with sulfur atoms bound to the gold surface as thiolate species,^58,60−62^ whereas the doublet with binding energies of (164.1 ± 0.4) and (165.3 ± 0.4) eV for S 2p_3/2_ and S 2p_1/2_, respectively, is attributable to unbound thiols.^58,61^ The doublet with binding energies of (168.6 ± 0.3) and (169.8 ± 0.3) eV for S 2p_3/2_ and S 2p_1/2_, respectively, is assignable to oxidized sulfur species, formed upon exposure to air.^60,62,63^ Overall, laser made AuNPs showed stronger interactions with thiols and a higher content of bound sulfur species than conventionally synthesized citrate-capped–electroreduced AuNPs. In general, increasing the methylene chain length of the *n*-alkanethiols led to a higher ratio of unbound to bound thiols for the laser made AuNPs. The observed low amount of sulfur bound to gold at the conventionally synthesized citrate-capped–electroreduced AuNPs suggests weaker interactions between conventionally synthesized AuNPs and the thiols, which could be a result of site blocking by residual citrate molecules that remained at AuNP surface, even after electroreduction, due to incomplete citrate removal.

Although the ratio of surface gold to the sum of surface gold and carbon content can be derived from XPS data, its interpretation is complicated by several confounding factors. The C 1s signal is influenced not only by the underlying carbon support but also by the varying number of carbon atoms in the thiol molecules and the differing surface carbon contributions depending on the orientation of the thiols within the SAMs. In citrate-capped samples, residual citrate further complicates the signal. Deconvoluting these overlapping contributions is challenging. Therefore, we conclude that this ratio is too convoluted to serve as a reliable metric for meaningful interpretation.

### 2.4 CO_2_ Reduction at SAM–AuNP–hCFP Cathodes

**Figure 5:**
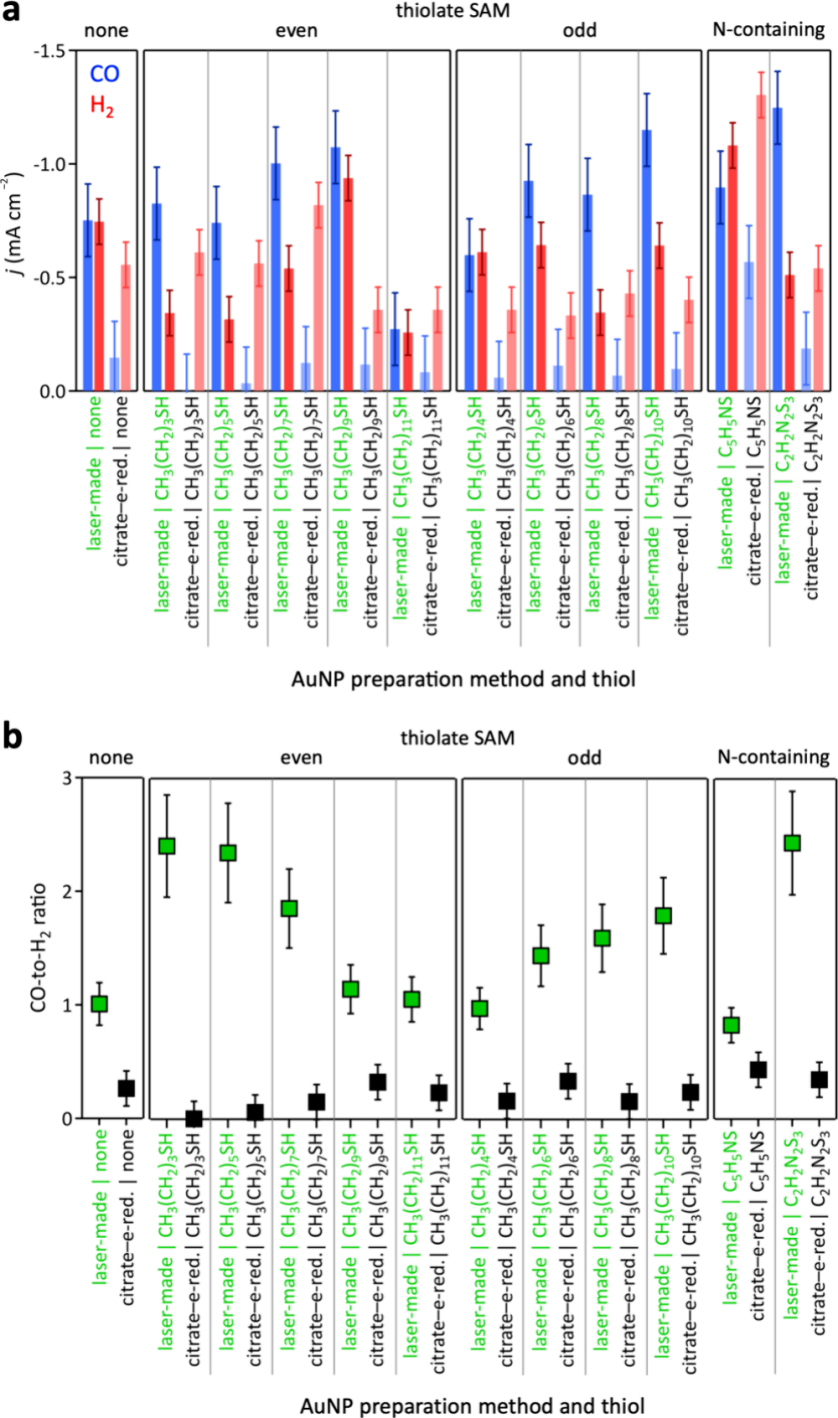
(a) Partial current densities for CO and H_2_ production and (b) CO-to-H_2_ ratios from aqueous CO_2_ reduction at −0.8 V vs RHE catalyzed by conventionally synthesized citrate-capped–electroreduced (black) or laser-made (green) AuNP–hCFP cathodes.

Electrocatalytic CO_2_ reduction data at integrated SAM functionalized laser-made or conventionally synthesized citrate-capped–electroreduced AuNP–hCFP cathodes are shown in Figure 5. In addition to the major products CO and H_2_, up to 1.3% formate was detected by NMR (Figure S8, Table S1), in agreement with reported data.^9,64,65^

Total current densities (Figures 5a and S9) were higher for laser-made than for analogous conventionally synthesized citrate-capped–electroreduced thiol functionalized AuNPs, likely due to a higher number of electrochemically active Au sites in the laser-made AuNPs (Figure S4). The CO-to-H_2_ ratio depended strongly on the nature of the thiolate and AuNP preparation method, with laser-made AuNPs exhibiting a higher CO-to-H_2_ ratio than conventionally synthesized citrate-capped–electroreduced AuNPs, irrespective of surface functionalization (Figure 5b). The CO-to-H_2_ ratios of conventionally synthesized citrate-capped–electroreduced thiol functionalized AuNPs were ≤0.44 with no trend discernible within error, likely due to residual citrate surfactants complicating the catalyst microenvironment. In contrast, the highest CO-to-H_2_ ratio of laser-made thiol functionalized AuNPs reached 2.44, with clear trends for even and odd *n*-alkanethiols as a function of methylene chain length, and for the nitrogen-containing thiols 4-mercaptopyridine and 2,5-dimercapto-1,3,4-thiadiazole (Figure 5b). Therefore, we focus in the following on laser-made thiol functionalized AuNPs.

Organic thiols form SAMs on gold surfaces.^66,67^ The driving force for self-assembly are hydrophobic van der Waals interactions between the methylene groups of the alkane chains.^16^ Therefore, longer-chain *n*-alkanes give rise to laterally more ordered SAMs.^16^ At planar gold surfaces, lateral order exists in *n*-alkane SAMs in two dimensions.^16^ The alkane chains of *n*-alkanethiolate SAMs are tilted with respect to the surface normal because this tilt maximizes the interchain interactions and lowers the overall surface energy on planar surfaces.^66^ Gold–thiolate bond formation is energetically favored at undercoordinated Au sites where Au atoms form significantly stronger bonds to S.^68,69^ At gold edges, lateral order between alkane chains can only form in one dimension along the chain of Au atoms, and *n*-alkanethiolate binding to gold corners eliminates lateral ordering. Mobility of thiolates on gold surfaces, especially at step edges, and on small AuNPs (2–4 nm core diameter) has been observed.^70^ Overall, the SAM structure on AuNPs is governed by the Au–S bond strength that depends on the coordination of gold sites and the interchain interactions, predicated on methylene chain length. As a result, short-chain *n*-alkanethiols, which possess a low driving force for lateral ordering, bind preferentially to undercoordinated gold sites, whereas the stronger interchain van der Waals interactions of longer-chain *n*-alkanethiols cause preferential binding to planar gold surfaces.

**Figure 6:**
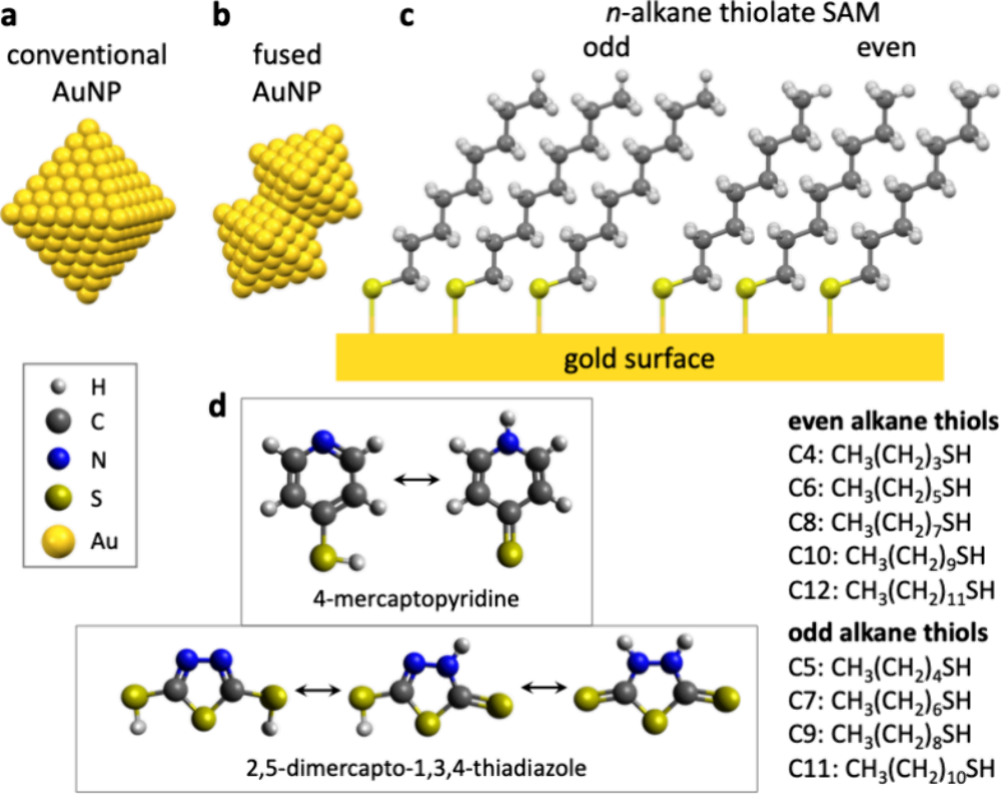
Schematics of (a) an equilibrium structure AuNP with plane, corner, and edge gold sites, and (b) a nonequilibrium AuNP, such as the laser-made cauliflower AuNPs of this work, illustrating the additional concave edge that emerges by nanoparticle fusing. (c) Molecular structures of odd and even *n*-alkanethiolate SAMs and (d) the nitrogen-containing thiols (with tautomers) used here. The *n*-alkanethiols of this work are listed on the right.

Methyl-terminated *n*-alkanethiolate SAMs provide hydrophobic surfaces,^17^ thus decreasing surface water and enhancing local CO_2_ concentrations. Dodecanethiolate SAMs on AuNPs, which were conventionally synthesized by a modified Brust method with tetraoctylammonium bromide and dodecanethiol surfactants,^71^ have been shown to act as a CO_2_-permeable membrane and enhance CO_2_ reduction selectivity and cathode stability.^23^ AuNPs prepared by equilibrium methods, such as the conventionally synthesized citrate-capped–electroreduced AuNPs used here, exhibit gold atoms in plane, convex corner, and convex edge sites (Figure 6a). Our cauliflower nonequilibrium laser-made AuNPs additionally possesses concave edges that emerged by fusing smaller AuNPs during the laser process (Figure 6b). Geometrically, a concave edge resembles the gold atoms under a one-dimensional string of gold atom vacancies. Organic thiolates do not bind to the gold atom under a surface vacancy, but form Au–S bonds at adjacent gold sites above the vacancy.^72^ As a result, organic thiolate SAMs do not bind to these concave edge Au sites, offering open gold sites for CO_2_ adsorption, corroborated by our finding of more electrochemically active Au sites in laser-made thiol-modified AuNPs (Figure S4), while the nearby SAMs simultaneously provide enhanced CO_2_ mass transport and beneficial elimination of microenvironment water. This explains our finding of higher CO-to-H_2_ ratios at laser-made AuNPs than at conventionally synthesized citrate-capped–electroreduced AuNPs (Figure 5).

While longer-chain *n*-alkanethiolate SAMs on gold exhibit slower charge transport than shorter-chain ones,^54^ and odd–even effects on charge transport have been observed as a result of differences in terminal group orientation, with even *n*-alkanethiolate SAMs giving higher current densities than the analogous odd series,^54,73,74^ charge transport through the SAM is unlikely to play a significant role in CO_2_ reduction and hydrogen evolution, as both reduction reactions occur at the gold surface.^75,76^ However, odd–even effects of *n*-alkanethiols influence the hydrophobicity of the outer thiolate ligand shell,^77^ which in turn affects CO_2_ mass transport. Additionally, the orientation of the terminal methyl group affects the packing of the SAM.^77^ In odd *n*-alkanethiolate SAMs, a methyl and a methylene group point upward, creating a higher surface hydrocarbon density per geometric area at the same thiolate coverage (Figure 6c), and ergo higher surface hydrophobicity, than the upward-oriented methyl groups of even *n*-alkanethiolate SAMs.^77^ Surfaces with higher hydrophobicity enhance the local CO_2_ concentration at the cathode while the amount of accessible surface water molecules is decreased.^78^

To rule out the influence of variation in *n*-alkanethiolate ligand coverage on the observed correlations, we quantified the thiolate-to-gold ratio for laser-fabricated AuNPs functionalized with *n*-alkanethiols. Normalization of the Au 4f_5/2_ signal of Au^+^, indicative of Au–S bonding,^47^ to the Au^0^ 4f_5/2_ signal provides a quantitative measure of the relative surface coverage of gold with *n*-alkanethiolate ligands. This thiolate-to-gold ratio was derived from the Au 4f signal due to its higher intensity compared to the S 2p signal, resulting in an improved signal-to-noise ratio. We specifically analyzed the 4f_5/2_ spin–orbit component instead of the Au 4f_7/2_ signal, as the former is less affected by overlap with higher binding energy signals, enabling more reliable interpretation. Our analysis revealed no significant variation in the thiolate-to-gold ratio across the series of *n*-alkanethiols (Figure S10).

Both CO_2_ reduction and hydrogen evolution are proton-dependent processes in aqueous media.^5,18,43,79^ Hydrophobic environments at the electrode–electrolyte interface can hinder proton transport by limiting the presence of interfacial water molecules, which are essential for proton mobility via the Grotthuss mechanism.^80^ In highly hydrophobic ligand shells, restricted water structuring and reduced hydrogen bonding networks can lead to decreased local proton availability and mobility,^81^ thereby hampering proton-dependent processes. Complicating matters further, protons are cations, whereas CO_2_ is charge-neutral; thus, the dominant mode of proton transport in the electrode microenvironment is drift within the cathodic electric field, rather than diffusion as in the case of uncharged species.^82^ The drift rate is governed by the electric field gradient in the interfacial region. This gradient is not static throughout the electrocatalyzed reaction and is influenced by several factors, including the distance from the electrode surface, electrolyte composition and ionic strength, local pH, interfacial solvation dynamics, and the chemical identity of surface-bound molecules, which can screen the electric field depending on their dielectric properties, dipole orientation, and thickness.^83^ As a result, both CO and H_2_ production are influenced by the *n*-alkanethiolate ligands, albeit to varying degrees.

Additionally, the extent of lateral ordering of *n*-alkanethiolates within SAMs^16^ is important for both CO_2_ reduction and H_2_ evolution, as both reactions proceed more readily at undercoordinated edge or corner sites. While literature on gold-catalyzed H_2_ evolution is limited, studies on platinum nanocatalysts indicate that edge sites dominate the H_2_ evolution reaction.^84^ Similarly, CO_2_ reduction preferentially occurs at undercoordinated gold sites.^40,85^

Partial current densities for hydrogen evolution at laser-made AuNPs functionalized with even *n*-alkanethiols were similar for C4, C6, and C12 SAMs, whereas C8- and C10-functionalized AuNPs exhibited elevated H_2_ evolution (Figure 5a). A similar trend was observed for odd *n*-alkanethiols: the partial current densities for H_2_ formation with C5, C7, and C11 SAMs were comparable within experimental error, though consistently higher than those of even *n*-alkanethiolate SAMs with similar chain lengths. In contrast, the C9 SAM exhibited a slightly lower H_2_ partial current density (Figure 5a). The reduced H_2_ evolution observed for C4- and C6-functionalized AuNPs is attributed to decreased lateral order within the SAM due to shorter methylene chain lengths and consequently increased preferential thiolate binding at undercoordinated gold sites. In contrast, increased lateral ordering at C8 and C10 SAMs correlates with enhanced H_2_ production. The reduced H_2_ and CO production observed with the C12 SAM–AuNPs suggests that mass transport limitations through the longest-chain SAM limited both reduction reactions.

Odd *n*-alkanethiolate SAMs exhibit higher surface hydrophobicity and lower lateral ordering compared to their even counterparts, due to the orientation of the terminal methyl groups.^77^ Reduced lateral ordering facilitates mass transport through the SAM but also promotes thiolate binding at undercoordinated gold sites. High hydrophobicity enhances CO_2_ mass transport but can hinder proton transport. The C5 and C7 SAMs displayed H_2_ partial current densities comparable to that of the C8 SAM (Figure 5a), suggesting that the effect of improved mass transport can offset limitations in proton availability and gold active site accessibility. The C9 SAM exhibited a lower H_2_ partial current density, comparable to that of the C4, C6, and C12 SAMs, likely due to its higher hydrophobicity and hindered mass transport through this longer-chain odd *n*-alkanethiolate SAM. In contrast, the C11 SAM displayed a similar H_2_ partial current density to the C5 and C7 SAMs, suggesting that increased lateral order and the associated greater availability of undercoordinated gold sites played a dominant role in governing H_2_ evolution. This interpretation is supported by the high CO production observed for C11-functionalized AuNPs.

The CO partial current densities for even *n*-alkanethiolate SAMs (C4, C6, C8, and C10) were similarly high, whereas CO generation at the C12 SAM was markedly lower (Figure 5a). This decrease is attributed to restricted mass transport through the longest-chain even *n*-alkanethiolate SAM, likely due to the increased distance between the outer ligand shell and the gold surface, as well as the lower surface hydrophobicity of even compared to odd *n*-alkanethiolate SAMs. In contrast, the CO partial current densities for odd *n*-alkanethiolate SAMs (C5, C7, C9, and C11) increased steadily with methylene chain length (Figure 5a), suggesting that the chain-length-dependent increase in internal hydrophobicity, combined with the intrinsically higher surface hydrophobicity of odd relative to even *n*-alkanethiolate SAMs, enhanced CO_2_ mass transport, while improved lateral order facilitated access to catalytically active gold sites.

**Figure 7:**
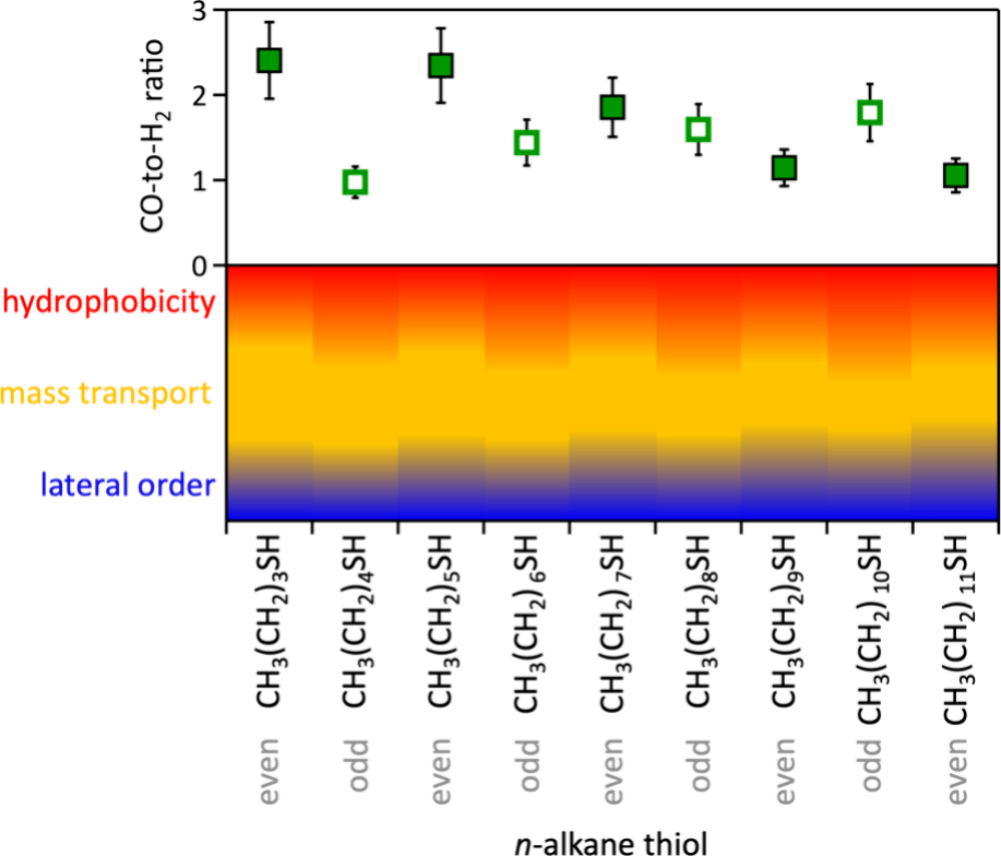
Schematic illustration of the key factors by which surface-bound *n*-alkanethiols influence the CO-to-H_2_ ratio during aqueous CO_2_ reduction at −0.8 V vs RHE, catalyzed by laser-synthesized AuNPs functionalized with *n*-alkanethiolate SAMs. Arbitrary units reflecting observed experimental trends were assigned to hydrophobicity, mass transport, and lateral order. The CO-to-H_2_ ratios correspond to the data presented in Figure 5; solid squares, even *n*-alkanethiolate SAMs, open squares, odd *n*-alkanethiolate SAMs.

The CO-to-H_2_ ratio, shown in Figure 5b, is a measure for the competition of the CO and H_2_ evolution rates. It reflects the influence of methylene chain length, terminal methyl group orientation in even versus odd *n*-alkanethiolate SAMs, and the resulting effects on surface and interchain hydrophobicity, as well as CO_2_ and proton mass transport. Additionally, lateral ordering impacts gold active site accessibility, further contributing to the observed trends in CO and H_2_ production. The competing effects and trends are visualized in Figure 7. The CO-to-H_2_ ratio shows clear trends with respect to even and odd *n*-alkanethiolate SAMs. The CO-to-H_2_ ratio of the C12 thiolate on gold was 1.1, similar to that of unfunctionalized laser-made AuNPs, which had a CO-to-H_2_ ratio of 1.0. The highest observed CO-to-H_2_ ratio was 2.4 at C4 and C6 SAM-functionalized AuNPs (Figure 5b).

Odd–even effects, arising from the orientation of terminal methyl groups, were more pronounced in AuNPs functionalized with shorter-chain *n*-alkanethiolate SAMs. Compared to longer-chain SAMs, these shorter-chain analogues exhibit enhanced mass transport through the thinner SAM layer and weaker interchain van der Waals interactions, resulting in reduced lateral order and lower internal hydrophobicity.^86,87^ We observed a significantly higher CO-to-H_2_ ratio in AuNPs modified with even short-chain *n*-alkanethiolate SAMs (Figure 7), primarily due to suppressed H_2_ evolution rather than increased CO production (Figure 5a). This suggests that the upward orientation of the terminal methyl group in even short-chain *n*-alkanethiolate SAMs, along with the associated greater lateral order and higher interchain hydrophobicity compared to odd SAMs, plays a more critical role in enhancing the CO-to-H_2_ ratio, likely by reducing the local presence of water at the gold surface. Additionally, preferential binding of laterally more ordered even *n*-alkanethiolates to planar gold surfaces likely leaves a greater number of CO_2_ reduction-active edge and corner sites accessible compared to odd short-chain *n*-alkanethiolate SAMs. In contrast, the increased outer ligand shell hydrophobicity of odd *n*-alkanethiolate SAMs^66,77^ and the resulting enhancement in CO_2_ mass transport appear to be less influential.

Conversely, with increasing methylene chain length, the CO-to-H_2_ ratio became higher for odd compared to even *n*-alkanethiolate SAMs, with a crossover occurring around C7–C9 (Figure 7), suggesting a shift in the dominant governing mechanism relative to that in short-chain systems. Chain-length-dependent structural changes toward increased lateral ordering have been reported for *n*-alkanethiolate SAMs on gold near this methylene chain length.^86,87^ The CO-to-H_2_ ratio was lower for AuNPs functionalized with longer-chain SAMs than for those modified with even short-chain analogues. Longer-chain SAMs exhibit reduced mass transport through the thicker monolayer, stronger interchain hydrophobic interactions and increased lateral order relative to their shorter-chain counterparts.^66^ For even *n*-alkanethiolate SAMs with more than nine methylene units, the CO and H_2_ partial current densities were identical within experimental error (Figure 5a), resulting in a CO-to-H_2_ ratio near unity. This indicates that the slower CO_2_ mass transport through the thicker SAM layer (relative to short-chain SAMs) limited competition with H_2_ evolution.

In contrast, a higher CO-to-H_2_ ratio was observed for odd long-chain *n*-alkanethiolate SAMs, suggesting that the increased outer shell hydrophobicity of odd SAMs governed the CO_2_ reduction activity in these systems. The low solubility of CO_2_ in aqueous electrolytes (33 mM at standard conditions) can lead to local depletion of CO_2_ near the catalyst surface, limiting CO_2_ reduction.^18^ Therefore, the increased hydrophobicity of the outer ligand shell in odd *n*-alkanethiolate SAMs, which attracts dissolved CO_2_, likely contributed to the improved CO-to-H_2_ ratio. Due to the stronger interchain interactions and enhanced structural rigidity of longer-chain *n*-alkanethiolate SAMs, the relative influence of terminal methyl group orientation is diminished, resulting in less pronounced odd–even effects (Figure 7).

**Figure 8:**
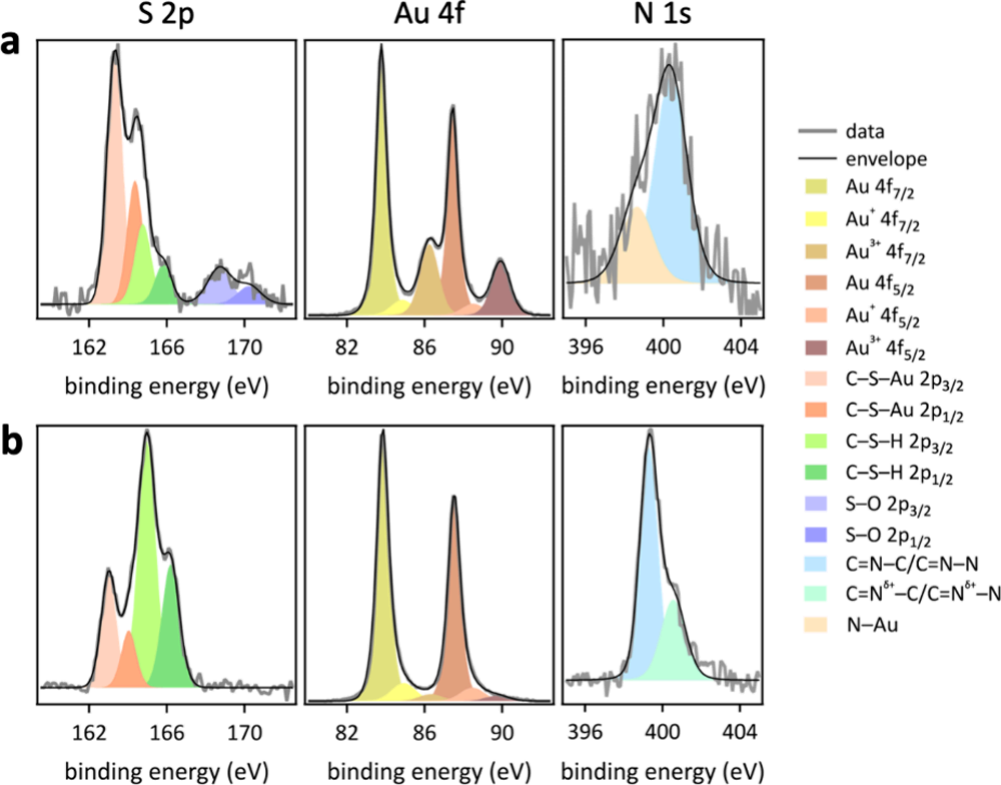
High-resolution S 2p, Au 4f, and N 1s XPS data of (a) 4-mercaptopyridine and (b) 2,5-dimercapto-1,3,4-thiadiazole functionalized laser-made AuNP–hCFP cathodes.

SAMs formed from nitrogen-containing organic thiols exhibit surface basicity,^88^ assisting in CO_2_ adsorption.^19^ Therefore, we functionalized AuNPs with 4-mercaptopyridine or 2,5-dimercapto-1,3,4-thiadiazole. Two tautomers exist for 4-mercaptopyridine, which delocalize the double bonds such that the pyridine tertiary amine is partially a secondary amine (Figure 6d). The resulting NH group can attract interfacial water that enhances undesired H_2_ evolution. At low pH, the surface structure of 4-mercaptopyridine SAM on Au(111) is upright and forms a dense phase.^89,90^ At neutral pH, such as the conditions of this study, 4-mercaptopyridine can form a disulfide dimer upon binding to gold,^55^ and gold binding through the 4-mercaptopyridine nitrogen group has been observed, indicative of a lying-flat binding motif.^56^ Among all laser-made AuNP cathodes, AuNPs functionalized by 4-mercaptopyridine exhibited the lowest CO-to-H_2_ ratio of 0.83 and the highest partial current density for H_2_, attributable to the lying-flat binding motif of 4-mercaptopyridine. High-resolution S 2p, Au 4f, and N 1s XPS data of 4-mercaptopyridine modified AuNP cathodes showed that 4-mercaptopyridine was bound to gold by sulfur and nitrogen atoms (Figure 8). An increased signal intensity for the Au 4f peaks at (87.0 ± 0.2) and (90.5 ± 0.2) eV, attributable to Au 4f_7/2_ and Au 4f_5/2_ of Au^3+^, respectively, was observed for 4-mercaptopyridine modified compared to *n*-alkanethiolate SAM functionalized laser-made AuNPs. In contrast, 2,5-dimercapto-1,3,4-thiadiazole modified laser-made AuNPs showed a negligible change of the Au 4f XPS signal, compared to *n*-alkane SAM functionalized laser made AuNPs (Figures 8 and S7). The observed shift in Au 4f XPS data of 4-mercaptopyridine modified laser-made AuNPs suggest an increased presence of electronegative species at the gold surface. Considering the structure of 4-mercaptopyridine (Figure 6), this electronegative species is likely nitrogen bound to the gold surface, evident from N 1s XPS data (Figure 8a). For 4-mercaptopyridine, two N 1s peaks, fitted with Gaussian–Lorentzian peaks, each with a fwhm of (1.5 ± 0.4) eV, were required to match the measured data (Figure 8a). The predominant N 1s peak was observed at (400.5 ± 0.2) eV, assignable to pyridine N atoms.^91−94^ Additionally, a weaker peak was observed at 398.6 eV, consistent with nitrogen atoms bound to gold,^56,95,96^ corroborating the lying-flat surface binding motif of 4-mercaptopyridine on laser-made AuNPs, which explains the exceptionally low charge transfer resistance observed in EIS data, which is inconsistent with electron transfer through an upright SAM (Figure S6).

For the other nitrogen-containing organic thiol, 2,5-dimercapto-1,3,4-thiadiazole, three tautomers have been reported, namely 1,3,4-thiadiazole-2,5-dithiol, 5-mercapto-1,3,4-thiadiazole-2(3H)-thione, and 1,3,4-thiadiazolidine-2,5-dithione,^97^ which possess zero, one, or two NH groups, respectively (Figure 6d). The 1,3,4-thiadiazole-2,5-dithiol tautomer is favored upon binding to AuNPs at neutral pH and binds through the S atoms.^57^ The dithiol tautomer possesses two nitrogen atoms that are not hydrogenated and, therefore, provide surface basicity without attracting interfacial water, explaining our observation of the highest partial current density, i.e. catalytic activity, for CO and the highest CO-to-H_2_ ratio of 2.44, a 3-fold increase over the 4-mercaptopyridine functionalized AuNPs (Figure 5).

For laser-made AuNPs functionalized with 2,5-dimercapto-1,3,4-thiadiazole, two peaks with binding energies of 399.3 and 400.1 eV were required to match the N 1s XPS data (Figure 8b), attributable to unmodified and partially protonated pyridine N atoms, respectively.^92−94^ This partial protonation is likely a result of the formation of hydrogen bonds with water, as pyridine has a high affinity toward water.^93,94^ In contrast to these laser-made AuNPs, conventionally synthesized citrate-capped–electroreduced AuNPs that were functionalized with 2,5-dimercapto-1,3,4-thiadiazole showed N 1s data with peaks at binding energies of 400.0 and 401.2 eV, which were assigned to unmodified and partially protonated pyridine N atoms.^89,98^ The shift to higher binding energies in the citrate-capped–electroreduced compared to laser made AuNPs is likely due to interactions between the 2,5-dimercapto-1,3,4-thiadiazole and remaining citrate molecules at the surfaces of conventionally synthesized, citrate-capped AuNPs, even after electroreduction, corroborating that citrate removal was incomplete. Interactions between oxygen atoms in citrate and nitrogen atoms in 2,5-dimercapto-1,3,4-thiadiazole shift the N 1s binding energies to higher values because oxygen is more electronegative than gold.^99^

The Au 4f spectra of 4-mercaptopyridine or 2,5-dimercapto-1,3,4-thiadiazole modified AuNPs required six peaks to match the measured data (Figures 8 and S7). In all spectra, the predominant species was metallic gold at a binding energy of (84.4 ± 0.1) eV for Au 4f_7/2_ and (88.0 ± 0.1) eV for Au 4f_5/2_.^9^ The remaining species were attributed to Au^+^ and Au^3+^. The peaks at (85.6 ± 0.2) and (89.0 ± 0.1) eV were assigned to Au 4f_7/2_ and Au 4f_5/2_ of Au^+^ in Au–S, respectively,^47^ whereas the peaks at (87.0 ± 0.2) and (90.5 ± 0.2) eV are attributable to Au 4f_7/2_ and Au 4f_5/2_ of Au^3+^ in Au_2_O_3_, respectively.^9,46,47^ Overall, the XPS data underpin the binding motifs of nitrogen-containing thiol modifications of AuNPs and their ramifications for the AuNP microenvironments and concomitantly observed CO-to-H_2_ ratios in AuNP-catalyzed CO_2_ reduction.

## 3 Conclusions

We showed that the absence of surfactants in laser-synthesized AuNPs is essential for understanding and steering CO_2_ reduction activity and product selectivity in aqueous CO_2_ reduction electrocatalysis at AuNP–hCFP cathodes. The chemical identity of interfacial molecules plays a critical role in modulating how the electrochemical microenvironment influences catalytic performance. Controlling this identity is essential for understanding how materials impact key design criteria in CO_2_ reduction electrocatalysis. Pulsed laser in liquid synthesis of AuNPs offered additional advantages by enabling the preparation of nonequilibrium cauliflower AuNPs that possessed concave edges, providing more undercoordinated sites than the analogous equilibrium material and giving rise to superior CO_2_ reduction performance by laser-made AuNPs. Furthermore, the citrate capping ligands of conventionally synthesized AuNPs were not completely removed by a separate electroreduction step reported to remove citrate ligands, which complicated the catalyst microenvironment and lowered the CO_2_ reduction activity and selectivity for CO.

Functionalization of laser-made and conventionally synthesized citrate-capped–electroreduced AuNPs with *n*-alkanethiols with nine different methylene chain lengths and with two nitrogen containing thiols established how alkane chain length, odd–even effects, and the presence of microenvironment nitrogen sites of gold–thiolate SAMs affected the CO_2_ reduction activity and selectivity for CO. Clear trends were only discernible in integrated cathodes with laser-made AuNPs, and CO_2_ reduction performance was superior compared to that of conventionally synthesized citrate-capped–electroreduced AuNPs for all surface modifications, indicating that the electrochemical citrate removal was incomplete. For laser-made AuNPs functionalized with even *n*-alkanethiols, the CO-to-H_2_ ratio decreased as a function of methylene chain length from 2.4 (1-butanethiol) to 1.1 (1-dodecanethiol). Laser-made AuNPs modified with odd *n*-alkanethiols showed the opposite trend. The CO-to-H_2_ ratio increased as a function of methylene chain length from 1.0 (1-pentanethiol) to 1.8 (1-undecanethiol), presumably because of the higher hydrophobicity of odd over even *n*-alkanethiolate SAMs. This hydrophobicity, which increases with the number of methylene groups, enhances the mass transport of CO_2_ and simultaneously reduces the amount of microenvironment water, thus synergistically increasing CO over H_2_ production. Since both CO_2_ reduction and H_2_ evolution are proton-dependent processes, multiple competing factors influence their activity. These include the methylene chain length and terminal methyl group orientation in odd versus even *n*-alkanethiolate SAMs, which affect interfacial hydrophobicity as well as CO_2_ and proton mass transport. Furthermore, the degree of lateral ordering within the SAM influences the accessibility of gold active sites, contributing to the observed trends in both CO and H_2_ production.

Surface functionalization with 4-mercaptopyridine resulted in the lowest CO-to-H_2_ ratio of 0.83 and the highest partial current density for H_2_ among all laser-made AuNP–hCFP cathodes, presumably because 4-mercaptopyridine did not form a SAM, evident from EIS data that showed exceptionally low charge transfer resistance, inconsistent with electron transfer through a SAM, and XPS data that showed Au–S and Au–N binding. Additionally, the secondary amine tautomer of 4-mercaptopyridine exhibits an NH group that attracts interfacial water, enhancing undesired H_2_ evolution. Functionalization of laser-made AuNP–hCFP cathodes with 2,5-dimercapto-1,3,4-thiadiazole produced the highest CO-to-H_2_ ratio of 2.4, three times more than the 4-mercaptopyridine modification. Additionally, the highest catalytic activity for CO was observed because the predominant dithiol tautomer forms a SAM and possesses two nitrogen atoms that are not hydrogenated and, therefore, provide surface basicity without attracting interfacial water.

Overall, our control over the AuNP catalyst microenvironment, achieved through pulsed laser in liquid synthesis of surfactant-free AuNPs and strategic functionalization with *n*-alkane and nitrogen containing thiols, steered product generation in aqueous CO_2_ reduction electrocatalysis. Guided by design principles targeting the suppression of hydrogen evolution, this approach offers key insights that establish a foundation for sustainable syngas production with tunable CO-to-H_2_ ratios.

## 4 Experimental Section

All chemicals were used as received. Deionized water with a resistivity of ≥17.5 MΩ·cm was obtained from a Thermo Scientific Barnstead Smart2Pure Pro UV/UF 15 LPH Water Purification System. The experiments were performed at room temperature and in ambient air. Glassware was cleaned with aqua regia, thoroughly rinsed with water, and dried before use. The error bars in the figures represent the standard deviations of triplicate measurements. Data analysis and graphing were conducted using Igor Pro 8.04 (Wavemetrics), unless otherwise stated.

### 4.1 Cathode Preparation

#### 4.1.1 Hydrophilic Carbon Fiber Paper

The process to prepare hCFP is described in detail elsewhere.^10^ Briefly, we modified the surfaces of carbon fiber paper, purchased from FuelCellStore (AvCarb MGL190, with 78% porosity), via sonication in a 1.0 M aqueous solution of sodium dodecyl sulfate (AG Scientific, ≥99%), followed by electrooxidation in a 0.1 M pH 8.7 aqueous KHCO_3_ (Alfa Aesar, 99.7–100.5%) at +1.63 V vs Ag/AgCl for 20 min.^10^

#### 4.1.2 Pulsed Laser in Liquid Synthesis of AuNPs

Laser-made AuNP colloids were obtained by pulsed laser in liquid synthesis, using a gold foil target (99.99%, 0.013″ thickness, 0.500″ diameter, Surepure Chemetals) and water. A 1064 nm, 8 ns pulsed laser beam obtained from a 10 Hz Nd:YAG laser (Spectra-Physics Quanta-Ray LAB-190), was used at a pulse energy of 50 mW. The beam was steered into a dual-axis galvanometer (Thorlabs) to scan the incident beam, which was focused by a 1064 nm antireflection-coated F-Theta lens with a 420 mm focal length (Eksma), across the surface of the solid target that was immersed in water. After 1 h of synthesis, 45 mL of AuNP colloid was obtained, with a concentration of 4.86 × 10^–3^ mg mL^–1^, determined by ICP-MS data.

#### 4.1.3 Preparation of AuNP–hCFP Cathodes

Working electrodes were prepared by depositing colloidal AuNPs, either laser-made (4.86 μg mL^–1^ in water) or commercial AuNPs (nanoComposix, 50 μg mL^–1^ in 2 mM aqueous sodium citrate), on hCFP, with dimensions of 2.4 cm (wide) × 3.8 cm (long). We note that during CO_2_ reduction experiments, only 5.8 cm^2^ of the geometric surface area was exposed to the electrolyte in the electrochemical H-cell. We placed the hCFP on the bottom of a custom-made Teflon tub, which was filled with aqueous AuNP colloid such that the hCFP was uniformly covered. The Teflon tub was filled with either 20.58 mL of laser-made AuNP colloid or 2 mL of citrate-capped AuNP colloid. The Teflon tub with the hCFP and the aqueous gold colloid was placed under a heat lamp at approximately 60 °C for 20 min to dry the liquid and yield the integrated electrode.

All AuNP–hCFP cathodes made with commercial, citrate-capped AuNP colloid were subjected to electroreduction at −1.28 V vs Ag/AgCl for 30 min in 0.1 M pH 8.7 aqueous KHCO_3_ electrolyte, followed by washing with copious amounts of water and drying in a dry nitrogen stream.^9^

#### 4.1.4 Functionalization of AuNPs with Organic Thiols

We used the organic thiols 1-butanethiol (Sigma-Aldrich, 99%), 1-pentanethiol (Sigma-Aldrich, 98%), 1-hexanethiol (Sigma-Aldrich, 95%), 1-heptanethiol (Thermo Scientific, 98%), 1-octanethiol (Sigma-Aldrich, ≥98.5%), 1-nonanethiol (Sigma-Aldrich, 99%), 1-decanethiol (Sigma-Aldrich, 96%), 1-undecanethiol (Sigma-Aldrich, 98%), 1-dodecanethiol (Sigma-Aldrich, ≥98%), 4-mercaptopyridine (TCI, >97%), or 2,5-dimercapto-1,3,4-thiadiazole (Sigma-Aldrich, 98%), to functionalize AuNPs. Integrated AuNP–hCFP cathodes were submerged in a 10 mM solution of each thiol in ethanol (Koptec, 200 proof) for 2 h. Functionalized AuNP–hCFP cathodes were subsequently washed with copious amounts of water and dried in a dry nitrogen stream. A glovebag (Spilfyter, Hands-in-Bag atmospheric chamber) purged with nitrogen (Airgas) was used for 4-mercaptopyridine.

### 4.2 Physical Characterization

#### 4.2.1 Dynamic Light Scattering Data

DLS data were collected to obtain AuNP sizes, using a Malvern Nano ZS instrument equipped with a 633 nm laser. Prior to data acquisition, colloidal gold nanoparticle samples (4.86 × 10^–3^ mg mL^–1^ in water) were sonicated for 5 min, before being transferred to a 1 mL quartz cuvette. Measurements were conducted at 25 °C with 2 min of equilibration time prior to data collection. A total of 9 measurements were collected for each sample, each averaging 11 runs with a duration of 10 s. Histograms for the size distribution of each sample were recorded.

#### 4.2.2 Inductively Coupled Plasma Mass Spectrometry Data

ICP-MS measurements were conducted at the University of Rochester Medical Center using a PerkinElmer NexION 2000 system, which features multielement detection and parts per billion/parts per trillion sensitivity. Gold was digested with aqua regia, prepared from concentrated Aristar Plus trace metal grade nitric acid (VWR, 69% w/w) and hydrochloric acid (VWR, 37% w/w). Additionally, Teflon tubs were filled to a height of 5 mm with aqua regia to assess residual gold remaining in the tub after the immersion deposition of gold colloid on hCFP.

#### 4.2.3 Optical Spectra

A fiber-optic ultraviolet to near-infrared optimized spectrometer (OCEAN-HDX-XR) and 1 cm path length quartz cuvettes were used. Air blanks were used, and a spectrum of water was collected and subtracted from the spectra of gold colloids.

#### 4.2.4 Raman Data

Samples containing aqueous colloidal laser-made AuNPs for Raman spectroscopy were concentrated using 1 mL of colloid in a 1.5 mL centrifuge tube (polypropylene, Eppendorf) and an Eppendorf Centrifuge 5418 at 14,000 rpm for 10 min, after which 666.7 μL of the supernatant was removed. The resulting concentrate was sonicated and had a concentration of 14.58 × 10^–3^ mg mL^–1^, determined by ICP-MS. Gold–thiolate samples were prepared by adding 2 μL of 1-butanethiol or 4 μL of 1-decanethiol to 400 μL of the concentrated aqueous AuNP colloid. Thiol control samples consisted of pure liquid 1-butanethiol or 1-decanethiol. Raman spectra were collected at room temperature using a Trivista 555 three-stage spectrometer equipped with a thermo-electrically cooled charge coupled device (Princeton Instruments PIXIS:400BR) and the last stage of a three-stage spectrograph (600 gr/mm, 100 μm slit width), resulting in a spectral resolution of 10 cm^–1^. A notch filter was installed to suppress the Rayleigh line because the estimated Raman vibration mode was above 300 cm^–1^, and the single monochromator collection with notch filter yields much higher signal collection efficiency than the triple monochromator collection. Cobalt Samba 150 lasers with wavelengths of 660 nm (60 mW) or 532 nm (100 mW) were focused on the sample, using an aspheric lens with 50 mm focal length. Data acquisition times were 53 min for thiol functionalized AuNPs with the 660 nm laser and 1 min for neat thiols with the 532 nm laser. Raman shifts were calibrated with cyclohexane. Raman spectra of thiol functionalized AuNPs were subjected to blank subtraction and polynomial baseline subtraction during data processing.

Density functional theory (DFT) calculations were performed to simulate Raman spectra. The thiol and gold–thiolate molecules were first optimized with the UFF force field using Avogadro.^100^ The DFT optimization and frequency analysis were performed at the wB97XD/def2-TZVP level of theory.

#### 4.2.5 Scanning Electron Microscopy Imaging

SEM images were obtained at UR-Nano, using a Zeiss Auriga scanning electron microscope, equipped with a Schottky field emission emitter, and operated at 20.00 kV with a working distance of 5.1 mm. Energy-dispersive X-ray (EDX) spectroscopy data were collected using an SEM-integrated EDAX Octane elect plus spectrometer with a with silicon drift detector.

#### 4.2.6 X-ray Photoelectron Spectroscopy Data

XPS data were collected using a Kratos Axis Ultra XPS instrument with a monochromatized Al Kα source. Samples were prepared by washing with water, drying, and affixing to double-sided adhesive copper tape. The instrument operated at 200 W and 15 kV under a base pressure of 3.0 × 10^–8^ mbar. Core level scans had a 0.1 eV step size, 260 ms dwell time, and 20 eV pass energy, averaged over 5 scans. Binding energies were calibrated using the adventitious C 1s peak at 284.8 eV.^101^ Data analysis was performed using Casa (Version 2.3.24) with Shirley background subtraction,^102^ Gaussian/Lorentzian peak fitting, and using instrument-specific atomic sensitivity factors.

#### 4.2.7 X-ray Diffraction Data

XRD data were acquired at the Chemical Analysis Lab at the Rochester Institute of Technology using a Bruker D8 ADVANCE diffractometer with Cu Kα radiation, with the tube operating at 40 kV and 40 mA. We used a 0.6 mm primary slit, 0.5 mm secondary slit, and 2.5 mm secondary antiscatter screen. Diffracted radiation was detected by a Lynxeye detector. The scan had a resolution of 0.025° in 2θ and a 1 s counting time per step, taking approximately 50 min per sample. Background subtraction was performed using the Bruker DIFFRAC.SUITE software.

#### 4.2.8 Zeta Potential

A Malvern Zetasizer Nano ZS instrument was used to measure the zeta potential. Prior to data collection, colloidal gold nanoparticle samples (4.86 × 10^–3^ mg mL^–1^ in water) were sonicated for 5 min, before being transferred to disposable folded capillary cell (Malvern, DTS1070). Measurements were conducted at 25 °C with 2 min of equilibration time prior to data collection. The Smoluchowski model was used to determine the zeta potential from the measured electrophoretic mobility. A total of 9 measurements per sample were collected with automatic data acquisition duration.

### 4.3 Carbon Dioxide Reduction

#### 4.3.1 Electrocatalysis

Data were collected analogous to details in ref (9). Briefly, a custom-made small-gap H-cell provided by the Jaramillo group at Stanford University was used.^52^ The electrolyte was CO_2_-saturated aqueous 0.1 M pH 6.8 KHCO_3_. The two compartments with 9 mL electrolyte each were separated by a Selemion anion exchange membrane (AMV-N). Both compartments were continuously sparged with humidified CO_2_ (Airgas, 99.99%) at room temperature for 20 min to ensure saturation of the electrolyte prior to electrocatalysis experiments. Calibrated mass flow controllers (Aalborg) maintained CO_2_ flow at 20 mL min^–1^. Integrated cathodes, consisting of bare or SAM functionalized AuNPs on hCFP, were used as working electrodes, the counter electrode was a Pt foil (Aldrich, 0.025 mm thick, 99.9%), and an Ag/AgCl (BaSi) reference electrode, calibrated against a reversible hydrogen electrode (RHE, Gaskatel HydroFlex) was used. The potentiostat (Bio-Logic, SP-150-EIS) enabled *iR* compensation (i, current, R, resistance) for solution resistance, as determined by impedance measurements using the built-in ZIR function.^9^ An automatic 85% *iR* compensation was used, with the remaining 15% manually corrected after data collection, in accordance with a published protocol.^52^ Chronoamperometry data were collected at −0.8 V vs RHE for 2.5 h. Gaseous CO_2_ reduction products were detected by an in-line gas chromatograph (GC, SRI, Multi-Gas #5 configuration) connected to the 2 mL headspace of the working electrode compartment of the electrochemical cell. Average faradaic efficiencies for a given experiment were obtained by averaging data points from 25 min forward, to exclude artifacts from solid-state material reductions.

#### 4.3.2 Quantification of Electrochemically Active Gold Atoms

Cyclic voltammograms were collected pre or post catalysis, scanning from +2.3 to −1.2 V vs RHE at 50 mV s^–1^ and analyzed using a method similar to that described in ref (103). Integrating the total charge under the Au^+^/Au^0^ reduction wave and dividing by the elementary charge gave the number of electrochemically active gold atoms.

#### 4.3.3 Electrochemical Impedance Spectroscopy Data

EIS data were acquired using the same electrochemical setup described as in the Electrocatalysis section above. EIS data were collected at an applied potential of −0.8 V vs RHE with AC-modulation at an amplitude of 5 mV, scanning from 200 kHz to 15 mHz, following a published protocol for collecting EIS data in an analogous electrochemical CO_2_ reduction cell as used here.^52^ To optimize the measurement time, single-sine excitation signal mode was employed from 200 kHz to 1 Hz, while multisine mode was employed from 1 Hz to 15 mHz. EIS spectra were fitted following a published protocol,^51^ utilizing the Z Fit feature of the Bio-Logic EC-Lab software. A Voigt circuit was used (Figure S6), which consisted of an electrolyte resistance (*R*_e_), a charge transfer resistance (*R*_ct_), a constant phase element related to double layer capacitance (*Q*_dl_), and an RQ pair accounting for the resistive and capacitive properties of the carbon fiber paper support (*R*_CFP_ and *Q*_CFP_). Constant phase elements were used to capture the nonideality of the capacity response.

#### 4.3.4 Quantification of Formate by NMR

^1^H NMR spectra were recorded at 500 MHz on a Bruker DPX-500 spectrometer at room temperature using a water presaturation method adjusted to the intensity of the water signal, with a 10% D_2_O and 90% H_2_O proportion, following a published protocol.^9^ A 90° width pulse was applied, followed by FID acquisition. The spectral width was 13.015 ppm (6510 Hz) centered on 4.826 ppm, with 32 kpt collected over 64 scans. The acquisition time was 2.5167 s. Spectral processing included phase correction, automatic baseline correction, and manual bias and slope corrections to ensure flat integrals on either side of peaks. Zero filling was applied by doubling the size of the FID before the Fourier transform. Samples were prepared by adding 35 μL of 35 mM DMSO (Fisher Chemical) and 50 mM phenol in D_2_O to 700 μL of electrolyte. Calibration curves for formate quantification were prepared using 6 samples with concentrations ranging from 50 mM to 1.6 μM. Peak integrals were normalized to the DMSO peak area.

## Supporting Information Available

Catalyst and electrolyte characterization data (PDF)

## Abbreviations

AuNP: gold nanoparticle
CFP: carbon fiber paper
DFT: density functional theory
DLS: dynamic light scattering
e-red: electroreduced
EDX: energy-dispersive X-ray spectroscopy
EIS: electrochemical impedance spectroscopy
fwhm: full width at half-maximum
GC: gas chromatography
hCFP: hydrophilic carbon fiber paper
ICP-MS: inductively coupled plasma mass spectrometry
NMR: nuclear magnetic resonance
Q: constant phase element capacitance
Q_dl_: constant phase element double layer capacitance
R: resistance
R_ct_: charge transfer resistance
R_el_: electrolyte resistance
RHE: reversible hydrogen electrode
SAM: self-assembled monolayer
SEM: scanning electron microscopy
XPS: X-ray photoelectron spectroscopy
XRD: X-ray diffraction
