# Supplementary material for: Interfacial Microenvironment Effects at Laser-Made Gold Nanoparticles Steer Carbon Dioxide Reduction Product Generation
Source: ACS Mater Au. 2025 Apr 29;5(3):522–36. doi: 10.1021/acsmaterialsau.4c00161 (PMC12082361; doi:10.1021/acsmaterialsau.4c00161)
Supplement: Supplementary file 1 — mg4c00161_si_001.pdf [file mg4c00161_si_001.pdf]

Supporting Information

Interfacial Microenvironment Effects at Laser Made Gold Nanoparticles Steer Carbon Dioxide Reduction Product Generation

Connor P. Cox,[†] *Qishen Lyu,*[‡] *Madeleine K. Wilsey,*[†] *Likun Cai,*[†] *Lydia R. Schultz,*[‡] *Jason R. Maher,*[‡] *Astrid M. Müller**[‡,†,§]

[†]Materials Science Program, University of Rochester, Rochester, New York 14627, United States.

[‡]Department of Chemical Engineering, University of Rochester, Rochester, New York 14627, United States.

[§]Department of Chemistry, University of Rochester, Rochester, New York 14627, United States.

*Email: astrid.mueller@rochester.edu

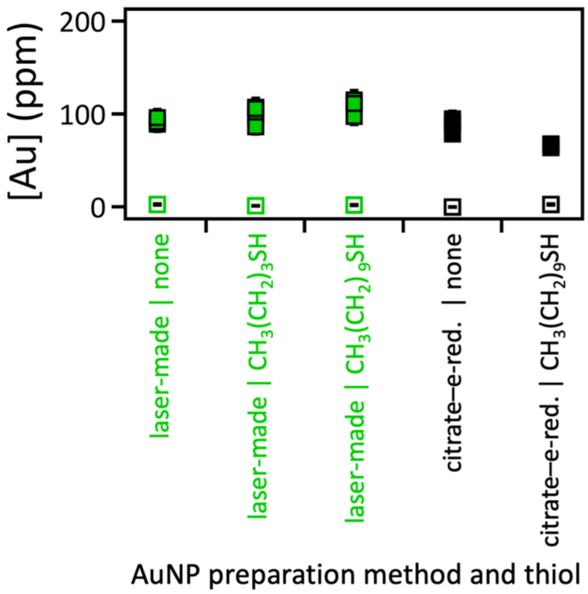

Figure S1. ICP-MS data of gold content of differently prepared AuNP–hCFP electrodes digested in aqua regia. The relative error is ± 9%.

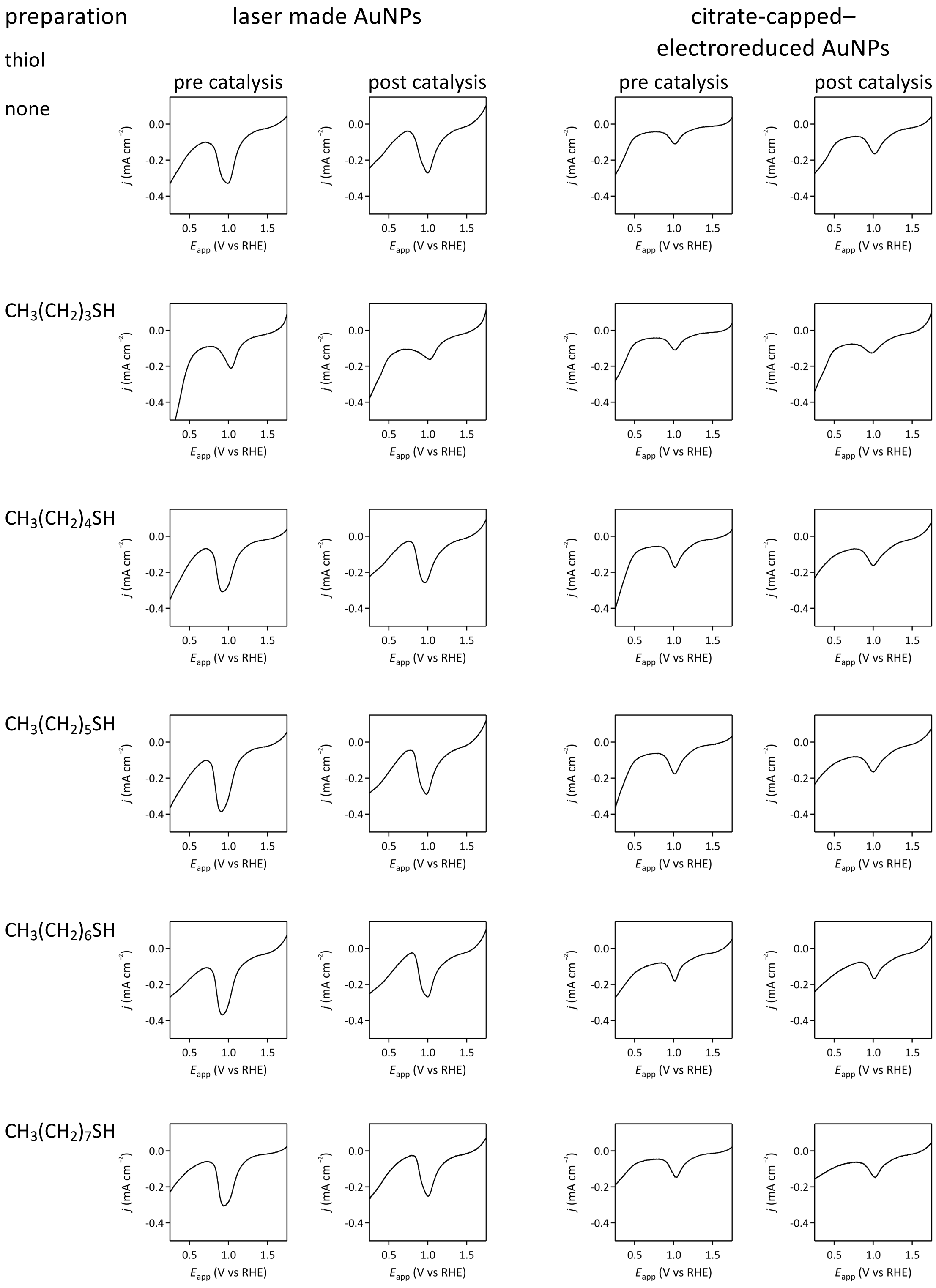

Figure S2. Cyclic voltammograms of the $Au^{I/0}$ reduction region.

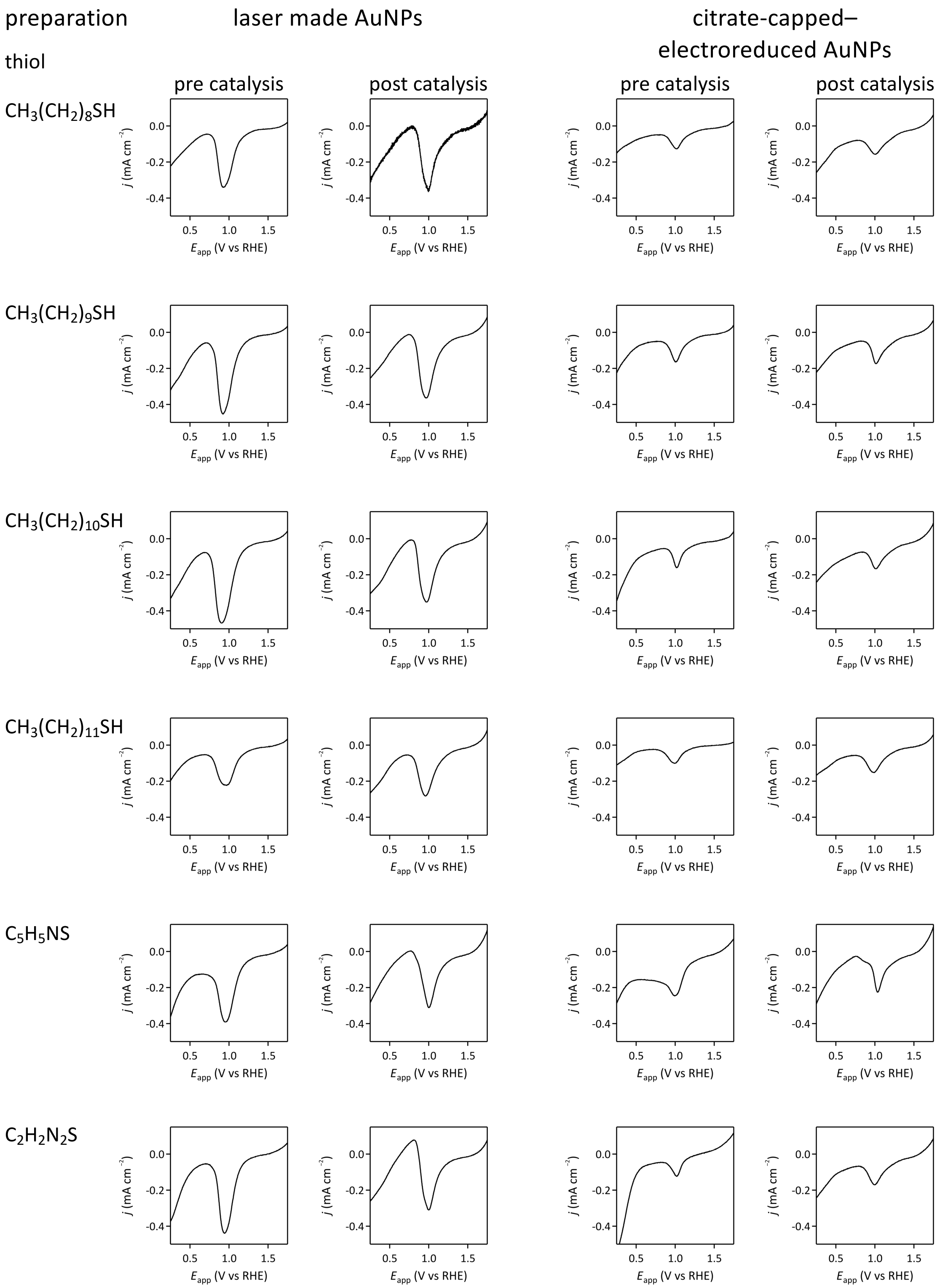

Figure S3. Cyclic voltammograms of the $Au^{I/0}$ reduction region.

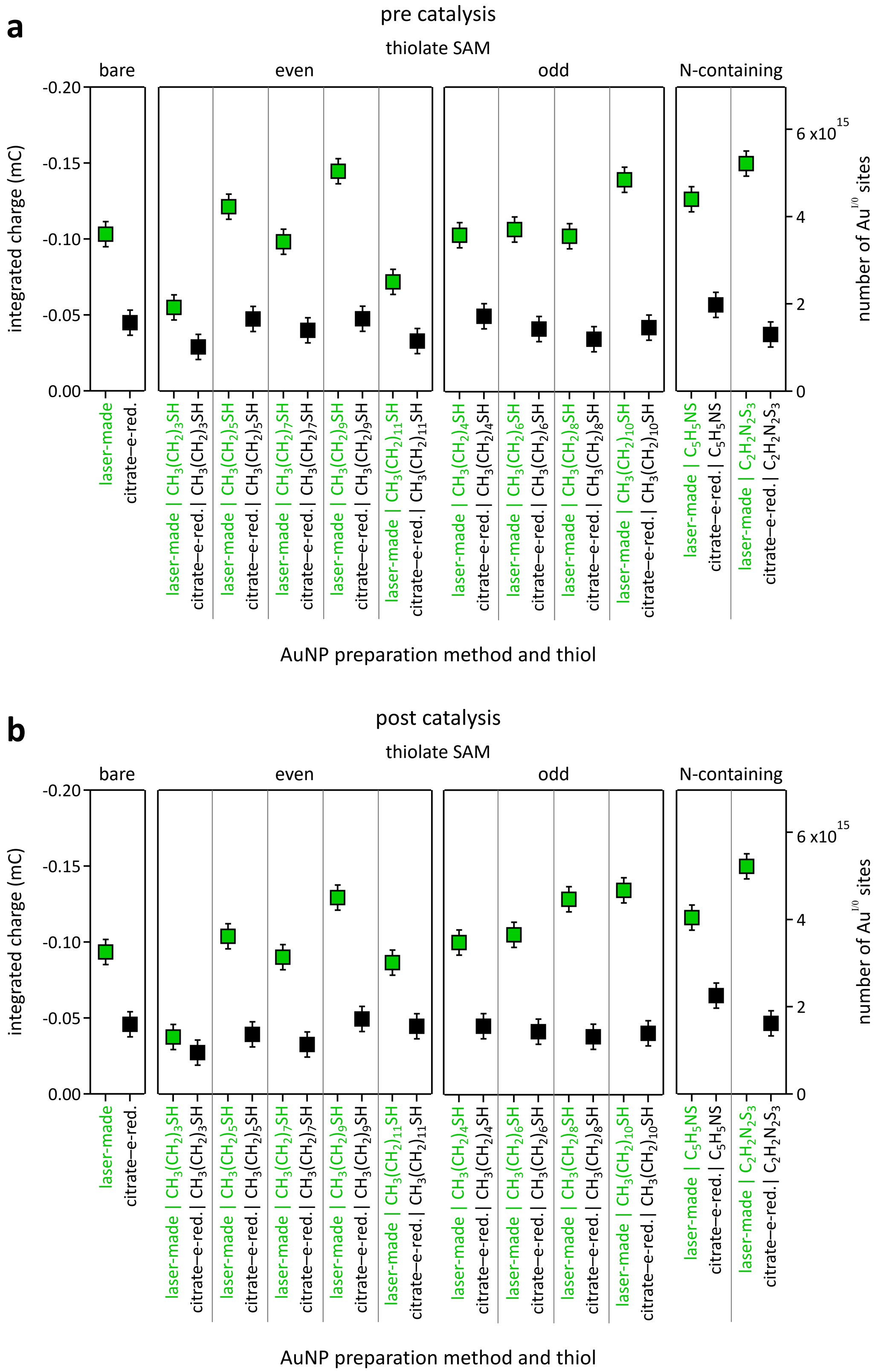

Figure S4. Number of accessible gold sites at conventionally synthesized citrate-capped–electroreduced (black) or laser-made surfactant-free (green) AuNP–hCFP cathodes (a) pre and (b) post electrocatalysis.

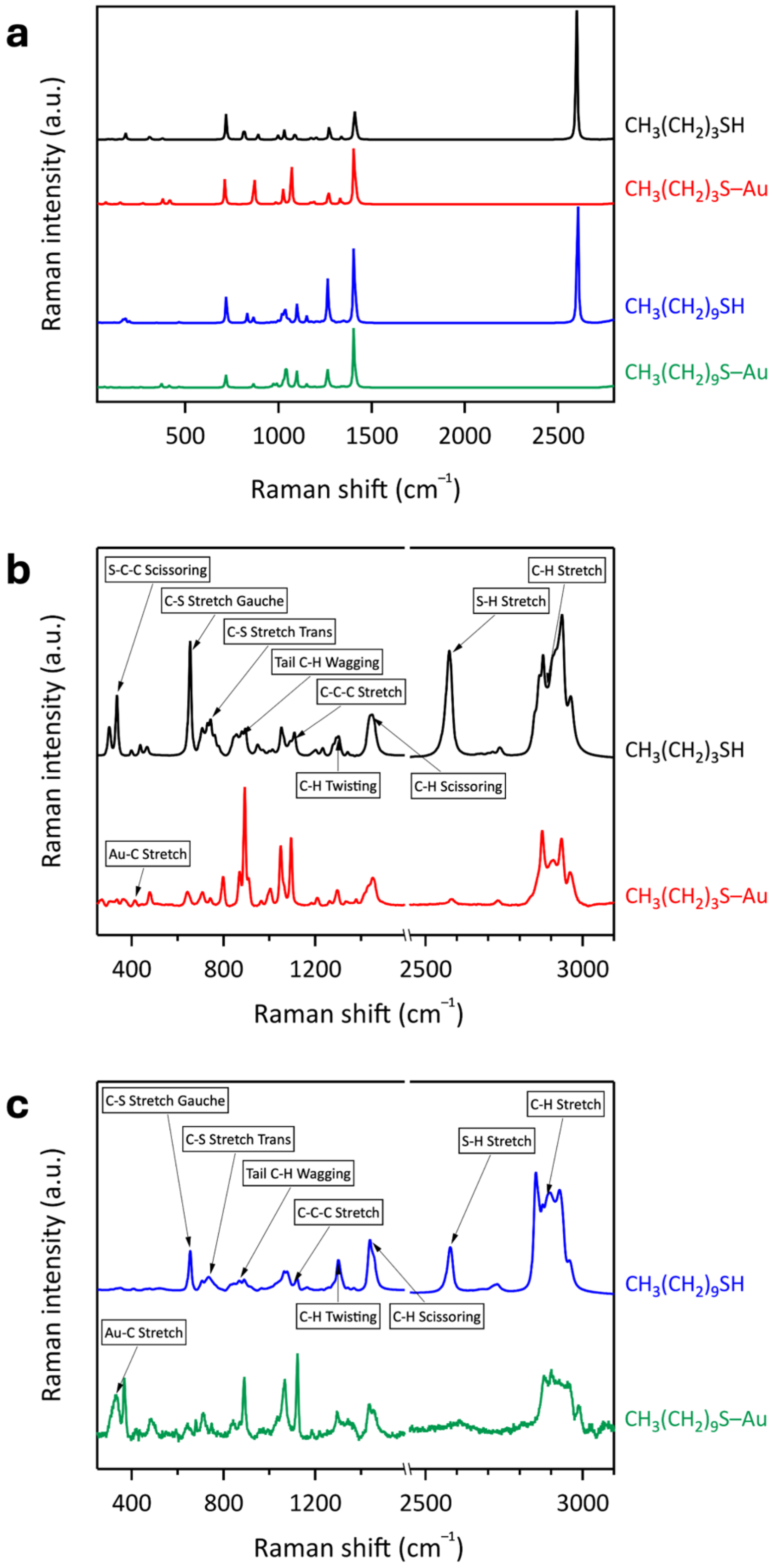

Figure S5. (a) Calculated Raman spectra. Measured Raman spectra with vibration assignments for thiols and laser-made AuNPs functionalized with (b) 1-butanethiol or (c) 1-decanethiol.

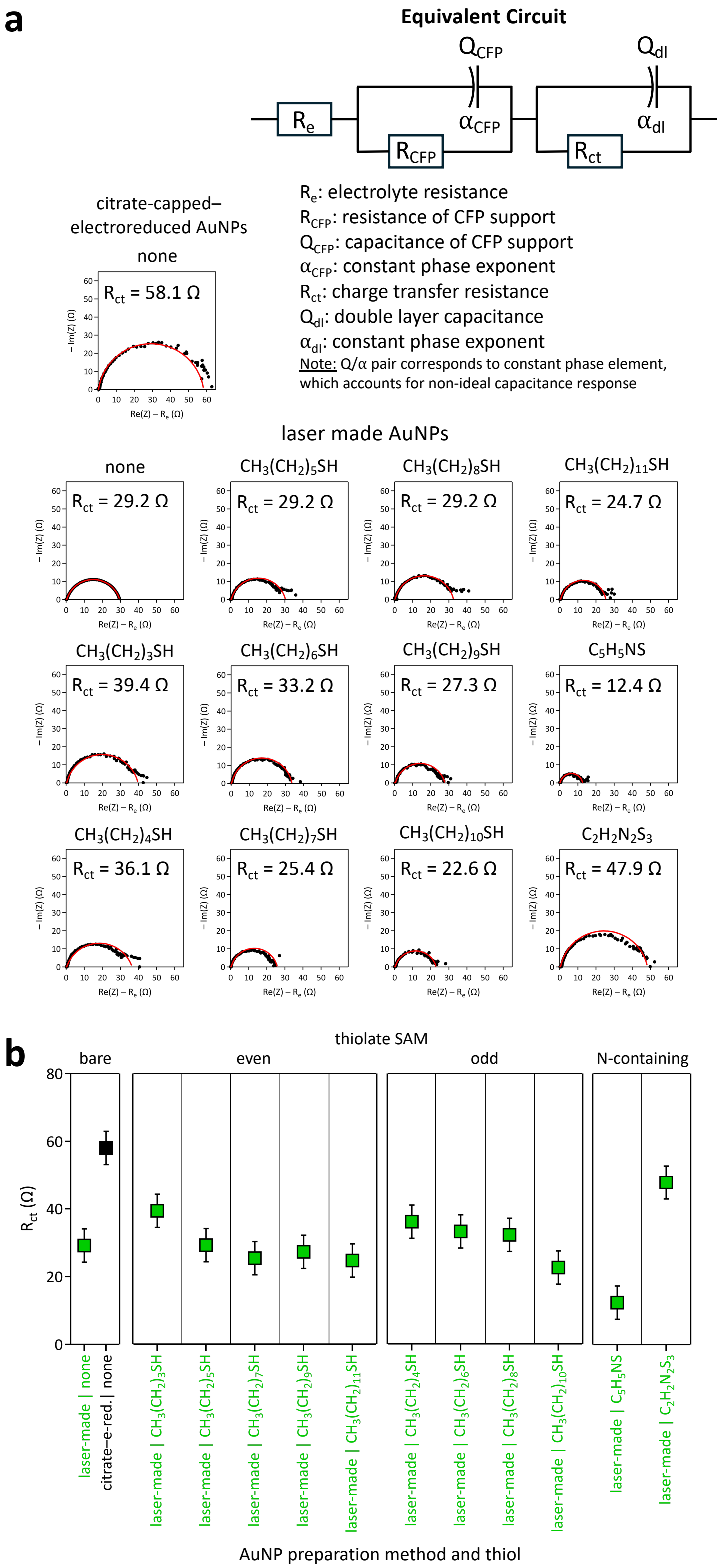

Figure S6. (a) Electrochemical impedance spectroscopy data (black circles) of bare and thiol functionalized AuNP–hCFP cathodes with fits (red lines) according to the depicted Randles equivalent circuit model. (b) Derived charge transfer resistance values.

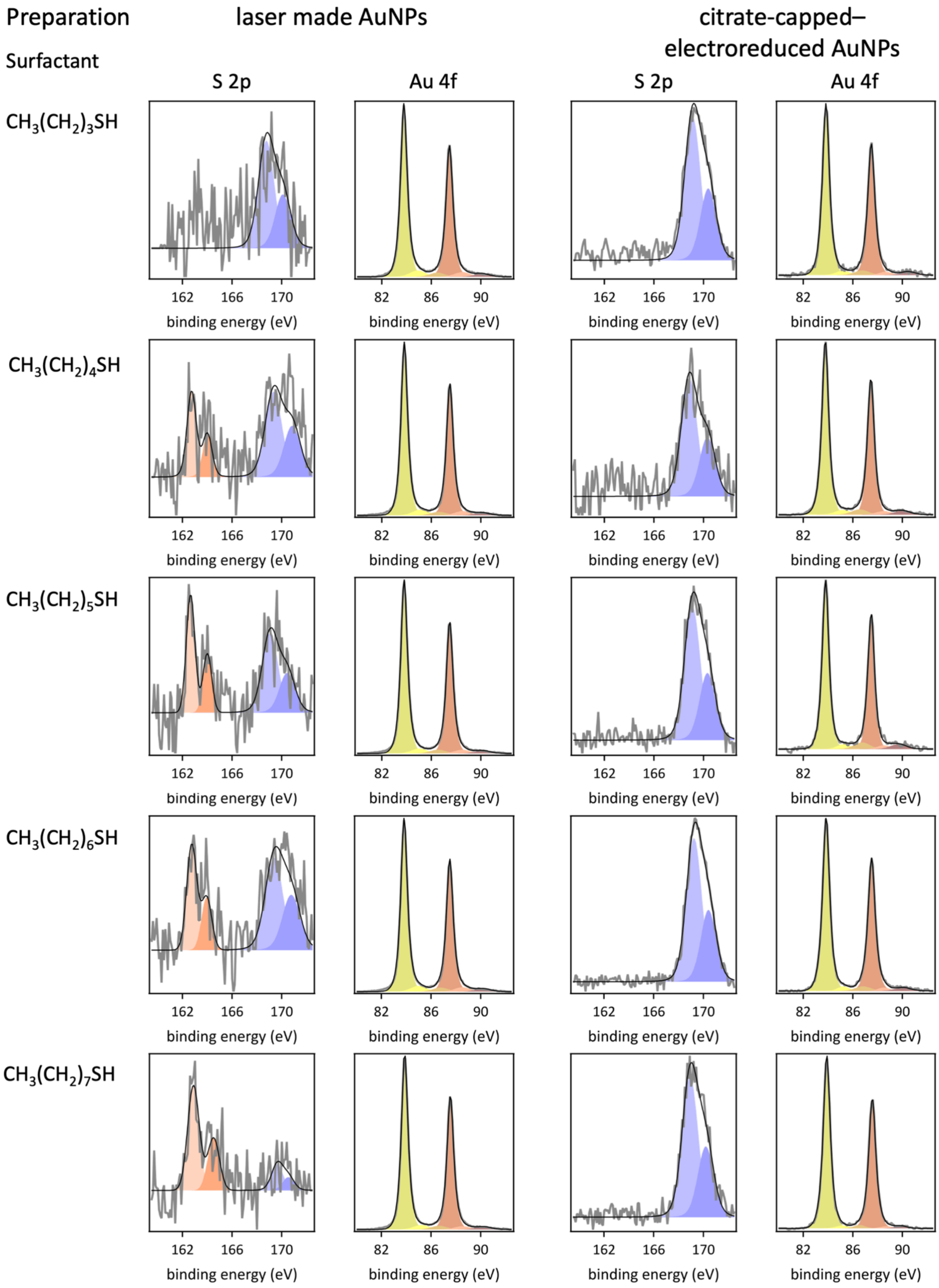

Preparation
laser made AuNPs
citrate-capped–
electroreduced AuNPs
Surfactant
S 2p
Au 4f
S 2p
Au 4f
$CH_3(CH_2)_3SH$
$CH_3(CH_2)_4SH$
$CH_3(CH_2)_5SH$
$CH_3(CH_2)_6SH$
$CH_3(CH_2)_7SH$
162
166
170
82
86
90
binding energy (eV)

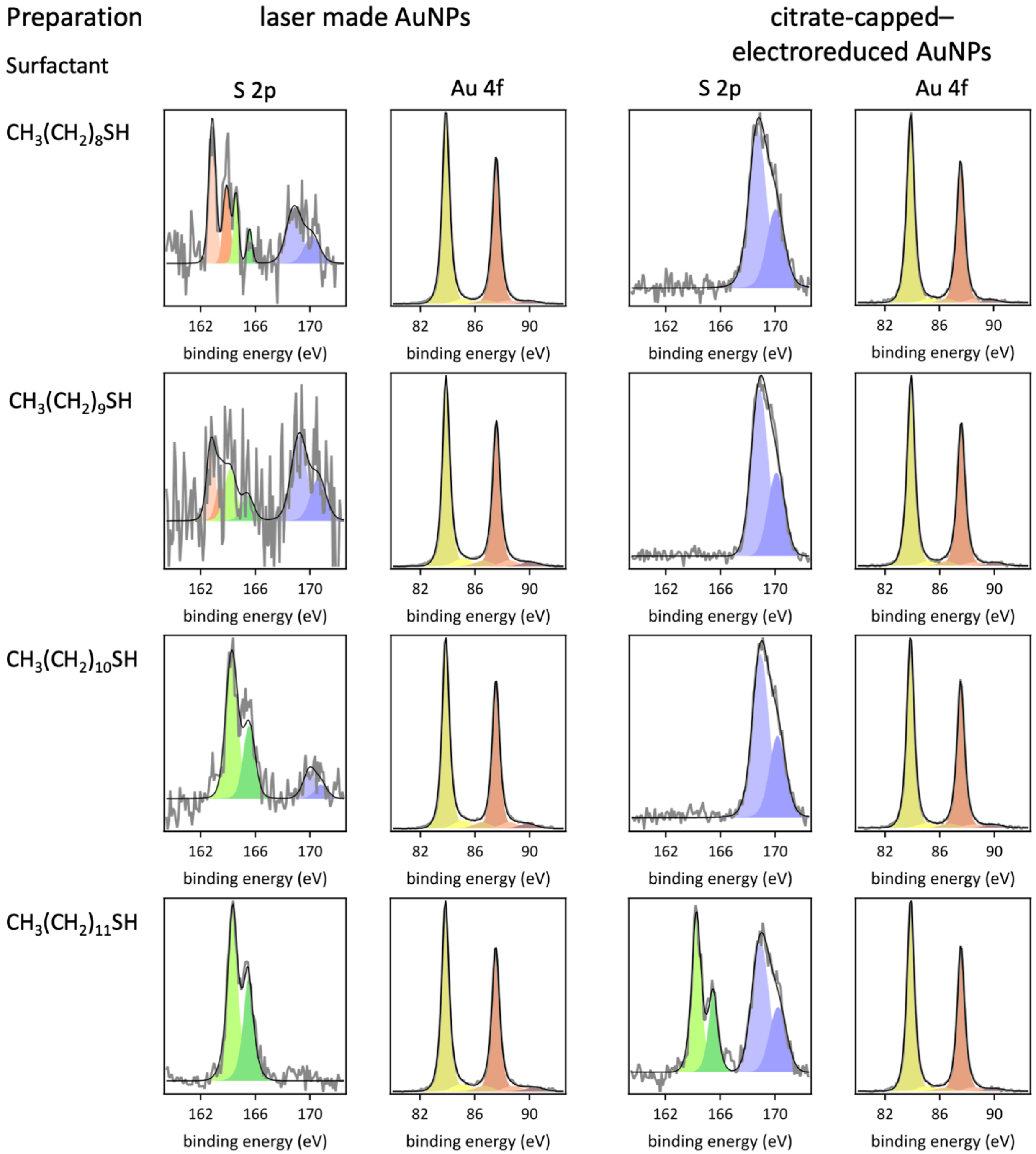
Preparation
laser made AuNPs
citrate-capped–
electroreduced AuNPs
Surfactant
S 2p
Au 4f
S 2p
Au 4f
CH3(CH2)8SH
CH3(CH2)9SH
CH3(CH2)10SH
CH3(CH2)11SH
162
166
170
82
86
90
binding energy (eV)

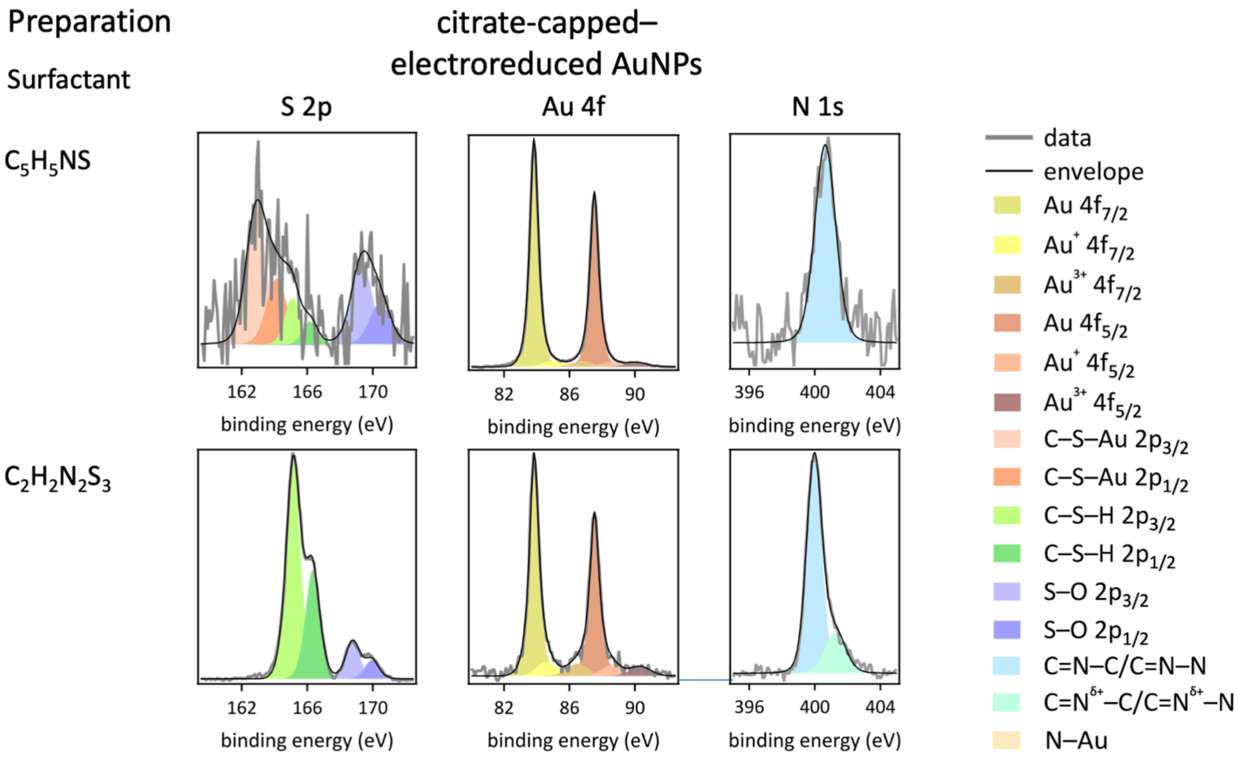

Figure S7. XPS data of thiol functionalized AuNP–hCFP cathodes.

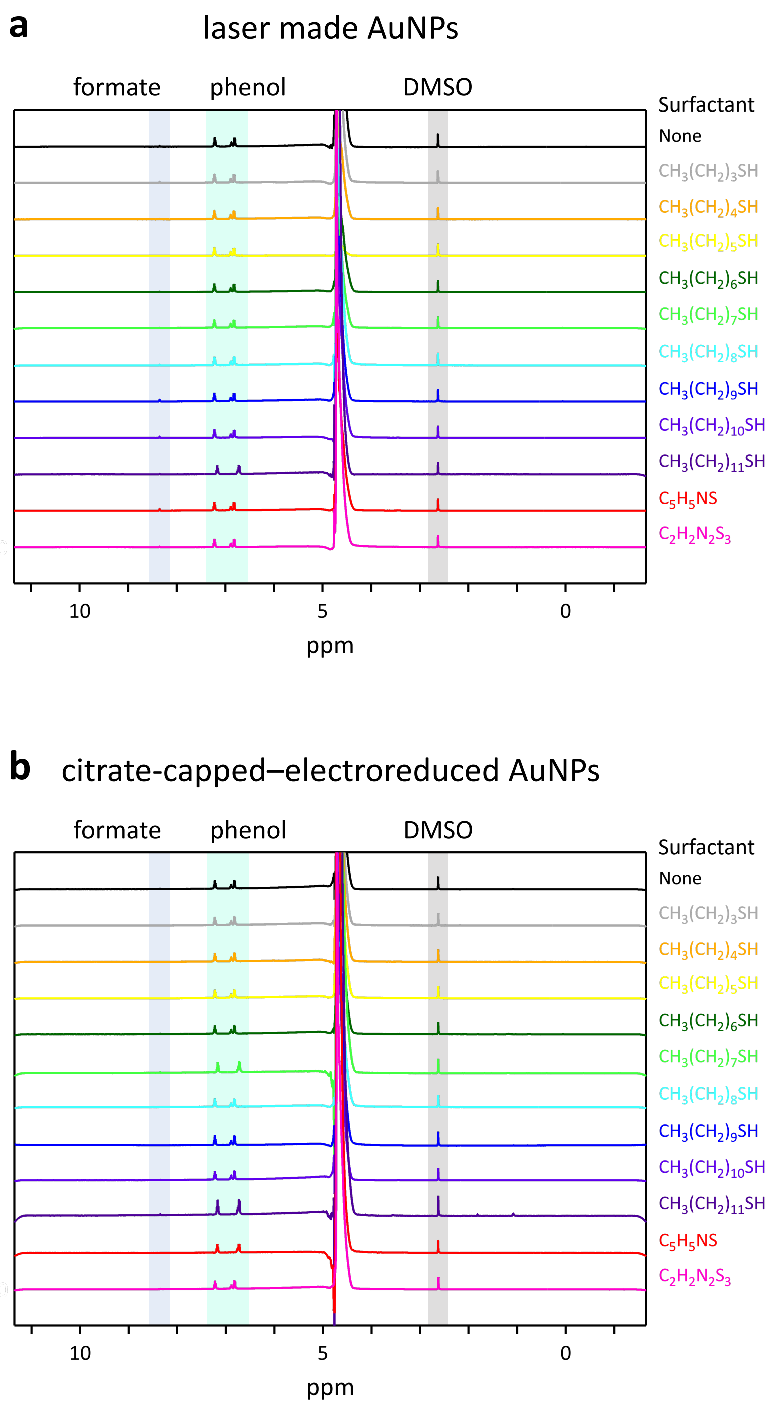

Figure S8. ^{1}H NMR spectra taken after constant potential electrocatalysis experiments of (a) laser-made or (b) conventionally synthesized citrate-capped–electroreduced AuNP–hCFP cathodes. Spectral regions corresponding to formate (blue), phenol (green), and DMSO (gray) are highlighted. Phenol and DMSO were added as internal standards to enable formate quantification.

Table S1. Faradaic efficiencies (FE) for formate, obtained from NMR data.

**Electrode preparation, surfactant**	**Formate FE (%)**
Laser-made, none	0.56
Laser-made, $CH_3(CH_2)_3SH$	0.80
Laser-made, $CH_3(CH_2)_4SH$	0.29
Laser-made, $CH_3(CH_2)_5SH$	0.05
Laser-made, $CH_3(CH_2)_6SH$	0.42
Laser-made, $CH_3(CH_2)_7SH$	0.16
Laser-made, $CH_3(CH_2)_8SH$	0.48
Laser-made, $CH_3(CH_2)_9SH$	1.01
Laser-made, $CH_3(CH_2)_{10}SH$	0.98
Laser-made, $CH_3(CH_2)_{11}SH$	1.11
Laser-made, C_5H_5NS	0.98
Laser-made, $C_2H_2N_2S_3$	0.53
Citrate-capped–electroreduced, none	0.53
Citrate-capped–electroreduced, $CH_3(CH_2)_3SH$	0.00
Citrate-capped–electroreduced, $CH_3(CH_2)_4SH$	0.00
Citrate-capped–electroreduced, $CH_3(CH_2)_5SH$	0.00
Citrate-capped–electroreduced, $CH_3(CH_2)_6SH$	1.31
Citrate-capped–electroreduced, $CH_3(CH_2)_7SH$	0.99
Citrate-capped–electroreduced, $CH_3(CH_2)_8SH$	0.00
Citrate-capped–electroreduced, $CH_3(CH_2)_9SH$	0.00
Citrate-capped–electroreduced, $CH_3(CH_2)_{10}SH$	0.35
Citrate-capped–electroreduced, $CH_3(CH_2)_{11}SH$	0.30
Citrate-capped–electroreduced, C_5H_5NS	0.00
Citrate-capped–electroreduced, $C_2H_2N_2S_3$	0.79

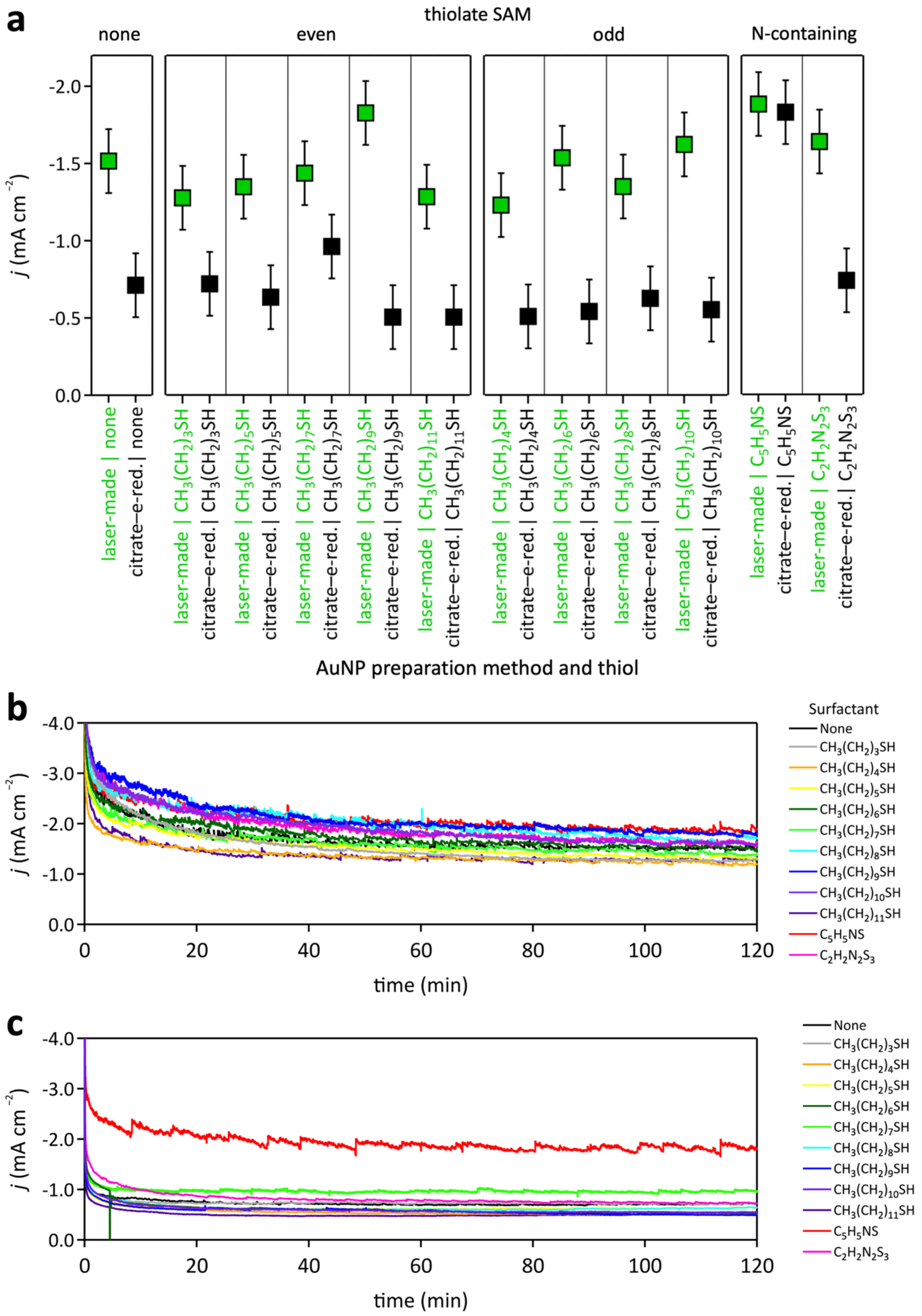

Figure S9. (a) Current densities and (b, c) chronoamperometry data for aqueous CO_2 reduction at –0.8 V vs RHE catalyzed by (b) laser-made or (c) conventionally synthesized citrate-capped–electroreduced AuNP–hCFP cathodes.

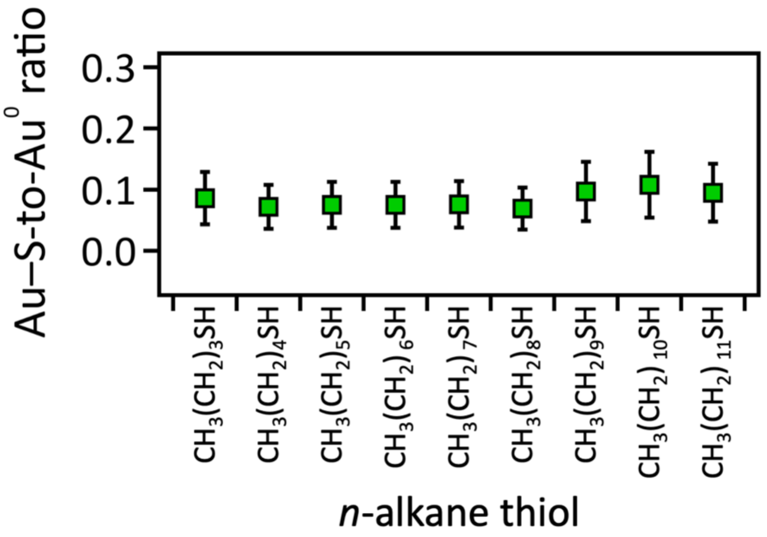

Figure S10. Ratio of Au–S to Au^0 signals of *n*-alkane thiol functionalized laser-made AuNPs, derived from the Au 4f XPS data shown in **Figure S7**.
